# Diverse high-affinity DNA aptamers for wild-type and B.1.1.7 SARS-CoV-2 spike proteins from a pre-structured DNA library

**DOI:** 10.1093/nar/gkab574

**Published:** 2021-07-07

**Authors:** Jiuxing Li, Zijie Zhang, Jimmy Gu, Hannah D Stacey, Jann C Ang, Alfredo Capretta, Carlos D M Filipe, Karen L Mossman, Cynthia Balion, Bruno J Salena, Deborah Yamamura, Leyla Soleymani, Matthew S Miller, John D Brennan, Yingfu Li

**Affiliations:** Department of Biochemistry and Biomedical Sciences, McMaster University, 1280 Main Street West, Hamilton, Ontario L8S 4K1, Canada; Department of Biochemistry and Biomedical Sciences, McMaster University, 1280 Main Street West, Hamilton, Ontario L8S 4K1, Canada; Department of Biochemistry and Biomedical Sciences, McMaster University, 1280 Main Street West, Hamilton, Ontario L8S 4K1, Canada; Department of Biochemistry and Biomedical Sciences, McMaster University, 1280 Main Street West, Hamilton, Ontario L8S 4K1, Canada; Michael G. DeGroote Institute for Infectious Disease Research, McMaster University, 1280 Main Street West, Hamilton, Ontario L8S 4L8, Canada; McMaster Immunology Research Centre, McMaster University, 1280 Main Street West, Hamilton, Ontario L8S 4K1, Canada; Department of Biochemistry and Biomedical Sciences, McMaster University, 1280 Main Street West, Hamilton, Ontario L8S 4K1, Canada; Michael G. DeGroote Institute for Infectious Disease Research, McMaster University, 1280 Main Street West, Hamilton, Ontario L8S 4L8, Canada; McMaster Immunology Research Centre, McMaster University, 1280 Main Street West, Hamilton, Ontario L8S 4K1, Canada; Department of Chemistry and Chemical Biology, McMaster University, 1280 Main Street West, Hamilton, Ontario L8S 4M1, Canada; Biointerfaces Institute, McMaster University, 1280 Main Street West, Hamilton, Ontario L8S 4L8, Canada; Michael G. DeGroote Institute for Infectious Disease Research, McMaster University, 1280 Main Street West, Hamilton, Ontario L8S 4L8, Canada; Department of Chemical Engineering, McMaster University, 1280 Main Street West, Hamilton, Ontario L8S 4L8, Canada; Department of Medicine, McMaster University, 1280 Main Street West, Hamilton, Ontario L8S 4K1, Canada; Michael G. DeGroote Institute for Infectious Disease Research, McMaster University, 1280 Main Street West, Hamilton, Ontario L8S 4L8, Canada; Department of Pathology and Molecular Medicine, McMaster University, 1280 Main Street West, Hamilton, Ontario L8S 4K1, Canada; Department of Medicine, McMaster University, 1280 Main Street West, Hamilton, Ontario L8S 4K1, Canada; Department of Pathology and Molecular Medicine, McMaster University, 1280 Main Street West, Hamilton, Ontario L8S 4K1, Canada; Michael G. DeGroote Institute for Infectious Disease Research, McMaster University, 1280 Main Street West, Hamilton, Ontario L8S 4L8, Canada; Department of Engineering Physics, McMaster University, 1280 Main Street West, Hamilton, Ontario L8S 4L7, Canada; School of Biomedical Engineering, McMaster University, 1280 Main Street West, Hamilton, Ontario L8S 4L8, Canada; Department of Biochemistry and Biomedical Sciences, McMaster University, 1280 Main Street West, Hamilton, Ontario L8S 4K1, Canada; Michael G. DeGroote Institute for Infectious Disease Research, McMaster University, 1280 Main Street West, Hamilton, Ontario L8S 4L8, Canada; McMaster Immunology Research Centre, McMaster University, 1280 Main Street West, Hamilton, Ontario L8S 4K1, Canada; Department of Chemistry and Chemical Biology, McMaster University, 1280 Main Street West, Hamilton, Ontario L8S 4M1, Canada; Biointerfaces Institute, McMaster University, 1280 Main Street West, Hamilton, Ontario L8S 4L8, Canada; Department of Biochemistry and Biomedical Sciences, McMaster University, 1280 Main Street West, Hamilton, Ontario L8S 4K1, Canada; Department of Chemistry and Chemical Biology, McMaster University, 1280 Main Street West, Hamilton, Ontario L8S 4M1, Canada; Biointerfaces Institute, McMaster University, 1280 Main Street West, Hamilton, Ontario L8S 4L8, Canada; Michael G. DeGroote Institute for Infectious Disease Research, McMaster University, 1280 Main Street West, Hamilton, Ontario L8S 4L8, Canada; School of Biomedical Engineering, McMaster University, 1280 Main Street West, Hamilton, Ontario L8S 4L8, Canada

## Abstract

We performed in vitro selection experiments to identify DNA aptamers for the S1 subunit of the SARS-CoV-2 spike protein (S1 protein). Using a pool of pre-structured random DNA sequences, we obtained over 100 candidate aptamers after 13 cycles of enrichment under progressively more stringent selection pressure. The top 10 sequences all exhibited strong binding to the S1 protein. Two aptamers, named MSA1 (*K*_d_ = 1.8 nM) and MSA5 (*K*_d_ = 2.7 nM), were assessed for binding to the heat-treated S1 protein, untreated S1 protein spiked into 50% human saliva and the trimeric spike protein of both the wildtype and the B.1.1.7 variant, demonstrating comparable affinities in all cases. MSA1 and MSA5 also recognized the pseudotyped lentivirus of SARS-CoV-2 with respective *K*_d_ values of 22.7 pM and 11.8 pM. Secondary structure prediction and sequence truncation experiments revealed that both MSA1 and MSA5 adopted a hairpin structure, which was the motif pre-designed into the original library. A colorimetric sandwich assay was developed using MSA1 as both the recognition element and detection element, which was capable of detecting the pseudotyped lentivirus in 50% saliva with a limit of detection of 400 fM, confirming the potential of these aptamers as diagnostic tools for COVID-19 detection.

## INTRODUCTION

The COVID-19 pandemic, caused by SARS-CoV-2 (severe acute respiratory syndrome coronavirus 2), has resulted in enormous loss of human life and economic burden to our society (1). The strategy of large-scale testing, contact tracing and isolation has been implemented by countries all over the world, which has been proven effective in controlling the spread of SARS-CoV-2. Although assays based on quantitative reverse-transcription real-time polymerase chain reaction (qRT-PCR) have been extremely successful for detecting SARS-CoV-2, the high cost and slow turnaround time prevent its use as a rapid screening tool for the general public (2). As countries around the world are working to slowly return to normalcy, the need for simpler, faster, more cost-effective large-scale testing has become critical to preventing or containing new outbreaks and helping create safe environments for social and economic activities (3).

Antigen (Ag) tests capable of detecting a protein unique to SARS-CoV-2 have been approved by various health agencies over the past year. Examples include the Abbott PanBio™ COVID-19 Ag test, the Abbott BINAX Now™ COVID-19 Ag test, and the Ellume rapid COVID-19 test, all of which operate as lateral flow immunoassays for detection of the nucleocapsid protein and utilize nasal pharyngeal swabs for sample collection. While relatively rapid and inexpensive, such antigen tests are inherently less sensitive than RT-PCR assays simply because the viral protein targets cannot be amplified. For example, a recent study found that the Abbott PanBio™ COVID-19 Ag test produced an overall sensitivity of 73.3% with symptomatic patients (4,5). In another study, the Abbott PanBio™ COVID-19 Ag test was examined for samples collected from assessment centers within the community using nasal pharyngeal (NP), throat, and saliva swabs. For NP sampling, the sensitivity and specificity were found to be 86.1% and 99.9%, respectively; however, throat sensitivity and saliva sensitivity were found to be only 57.7% and 2.6%, respectively (6). Even when using lower nasal swabs, the PanBio™ test still requires supervised self-swabbing for sample collection, and even then shows lower sensitivity than is obtained for nasal pharyngeal swab samples (7), highlighting the need for improved rapid tests with better clinical performance.

To improve the sensitivity and ease-of-use of rapid tests, one approach is to identify molecular recognition elements (MREs) with improved affinity and versatility relative to antibodies, which should allow detection of lower levels of virus in a wider range of sample matrixes. Aptamers, which are nucleic acid-based MREs that can be selected from random sequence pools by in vitro selection (8,9), can provide several advantages over antibodies for the development of rapid tests. These include small size, high chemical and thermal stability, easy and precise modification, scaleable production, minimal batch-to-batch variation (10), and the ability to apply aptamers to detect targets in a range of clinical samples (11–13), making aptamers popular MREs for biosensor development (14–16).

At this time, several groups have isolated DNA aptamers that recognize the Spike protein of wild-type SARS-CoV-2 (17–21). However, almost all these aptamers were selected to target the receptor-binding domain (RBD) of the spike protein for binding, with a goal of producing aptamers to inhibit the binding of the spike protein to the human angiotensin-converting enzyme 2 (ACE2). Thus far, no effort has been made to select aptamers using the full S1 subdomain of the spike protein as the binding target. However, targeting the full S1 subdomain may lead to the discovery of diverse high-affinity DNA aptamers that recognize different epitopes of this important protein.

Herein, we report on the selection of DNA aptamers against the wild-type monomeric S1 spike protein subdomain from a pre-structured random DNA library using a combination of selection methods based on magnetic bead-based separation and electrophoretic mobility shift assays (EMSA). The optimization of three high-affinity aptamers using truncation analysis to yield minimal sequences with nanomolar affinity for the S1 protein, and their performance for detection of the S1 protein, full trimeric spike protein and spike protein pseudotyped lentivirus both in buffer and diluted saliva is also reported. Finally, we demonstrate a simple saliva-based test for SARS-CoV-2 using the optimal S1 protein-binding aptamer.

## MATERIALS AND METHODS

### Chemicals and reagents

DNA oligonucleotides were ordered from Integrated DNA Technologies, purified by 10% (w/v) denaturing polyacrylamide gel electrophoresis with 8 M urea (dPAGE). The sequences of all the oligonucleotides used in this study are listed in Supplementary Table S1. Taq DNA polymerase was purchased from GenScript. The SARS-Cov-2 spike protein subunit S1 (catalog number: 40591-V08B1) and RBD (catalog number: 40592-V08B) was purchased from Sino Biological Inc. The SARS-CoV-2 full spike protein (molecular weight 140 kDa; plasmid encoding the mammalian cell codon optimized sequence for SARS-CoV-2 full length spike protein was generously gifted from the lab of Dr Florian Krammer, Ichan School of Medicine (22)) and the pseudotyped virus of SARS-CoV-2 (reagents were obtained through BEI resources, NIAID, NIH: SARS-Related Coronavirus 2, Wuhan-Hu-1 Spike-Pseudotyped Lentiviral Kit, NR-52948) were provided by the Miller lab at McMaster University. The viral concentration was determined by infecting the 293T-ACE2 cells (BEI NR-52511) with lentiviral particles carrying the luciferase gene and pseudotyped with the UKSARS-CoV-2 spike essentially as described by Crawford *et al.* (23). The expressed luciferase was measured by the bicinchoninic acid (BCA) assay and used to determine the viral concentrations. The full spike protein for the UK B.1.1.7 variant (UKS, catalog number: SPN-C52H6) was obtained from Acro Biosystems. The SARS-CoV-1 full spike protein (SARS1-S, catalog number: 100789–1) was purchased from BPS Bioscience Inc. The SARS-CoV-1 RBD (molecular weight: 30 kDa), MERS-CoV RBD (38 kDa) and HCoV-229E RBD (28 kDa) were provided by the Miller lab at McMaster University. The RBD proteins were generated in Expi293 cells and contained C-terminal His-tag for purification. The pooled human saliva (Lot 31887) was purchased from Innovative Research Inc (Novi, Michigan). Nitrocellulose blotting membranes (catalog no. 10600125) were purchased from GE Healthcare Inc. Nylon hybridization transfer membranes (NEF994001PK) were purchased from PerkinElmer Inc (Woodbridge, ON, Canada). Pierce™ streptavidin coated plates, streptavidin-conjugated HRP (Invitrogen™, catalog No. 19534-050), 1-Step™ Ultra TMB-ELISA Substrate Solution (Lot VL3152681), Ni-NTA magnetic agarose beads, T4 DNA ligase, T4 polynucleotide kinase (PNK), adenosine triphosphate (ATP), and deoxyribonucleoside 5′-triphosphates (dNTPs) were purchased from Thermo Scientific (Ottawa, ON, Canada). γ-[^32^P]-ATP was acquired from PerkinElmer. Bovine serum albumin (BSA), human thrombin and IgG from human serum were purchased from Sigma-Aldrich (Oakville, Canada). 4-(2-Hydroxyethyl)-1-piperazineethanesulfonic acid (HEPES), sodium chloride (NaCl), magnesium chloride (MgCl_2_), sodium phosphate dibasic (Na_2_HPO_4_), potassium phosphate monobasic (KH_2_PO_4_), potassium chloride (KCl), Tween-20, and all other chemicals were purchased from Sigma-Aldrich (Oakville, Canada) and used without further purification. Milli-Q water was used for all experiments.

### Conjugation of S1 protein to magnetic beads

HisPur Ni-NTA magnetic beads (16 μl, 5% w/v, 12.5 mg/ml) were first washed once with PBST buffer (0.5 ml, 1.8 mM KH_2_PO_4_, 10 mM Na_2_HPO_4_, 2.7 mM KCl, 137 mM NaCl, 0.01% v/v Tween-20). Magnetic beads pellets were then resuspended in 5× PBST buffer (40 μl). Imidazole (4 μl, 1 M), S1 protein with His-tag (100 μl, 0.5 mg/ml) and water (60 μl) were mixed with magnetic beads and incubated at 4°C for 12 h. Afterward, S1-conjugated magnetic beads (20 μl) were washed twice with PBST and resuspended in PBST buffer with 200 mM imidazole. The free S1 protein in the first wash fraction was collected. After heating at 90°C for 10 min, S1-conjugated magnetic beads were pelleted by a magnet, and the S1 protein in the supernatant was collected as the S1 protein bound on magnetic beads. The free and bound S1 proteins were analyzed by SDS PAGE. The S1 protein bound on magnetic beads was determined to be 0.147 mg/ml. The S1 protein-conjugated magnetic beads (1 mg/ml magnetic beads and 0.147 mg/ml S1 protein) were stored at 4°C and kept away from light before use.

### Selection of DNA aptamers for S1 proteins

The SELEX processes were carried out by a combination of magnetic bead-based and native gel-based methods. Briefly, the DNA library was diluted with Selection buffer (1× SB; 50 mM HEPES, 6 mM KCl, 150 mM NaCl, 2.5 mM CaCl_2_, 2.5 mM MgCl_2_, 0.01% v/v Tween-20, pH 7.4) and heated at 90°C for 1 min, followed by annealing at room temperature for 10 min. Then, the S1 protein-conjugated magnetic beads were washed twice with 1× SB and mixed with the DNA library at 23°C for 30 min. After washing three times with 1× SB (1 ml), the magnetic beads were resuspended with 1× Taq buffer (200 μl, 50 mM KCl, 10 mM Tris–HCl, 1.5 mM MgCl_2_, 1% v/v Triton X-100, pH 9.0) and heated at 90°C for 10 min. The DNA in the supernatant was collected, followed by the addition of the reverse primer RP1 (10 μl, 10 μM), the forward primer FP1 (10 μl, 10 μM), Taq DNA polymerase (2 μl, 5 U/μl), and dNTPs (20 μl, 2 mM) for PCR1. PCR1 was carried out using the following temperature profile: preheating at 94°C for 30 s; thermo cycles of 94°C for 30 s, 50°C for 30 s, and 72°C for 30 s; annealing at 72°C for 5 min. Next, the PCR1 product was used as the template for PCR2. The PCR2 mixture was prepared by mixing the PCR1 product (50 μl), FP1 (25 μl, 10 μM), RP2 (25 μl, 10 μM), 10× Taq buffer (50 μl), Taq DNA polymerase (5 μl, 5 U/μl), dNTPs (10 μl, 10 mM), and water (335 μl). The amplification reaction used the same temperature profile as PCR1. After amplification, the PCR2 product was pelleted by ethanol precipitation. Briefly, the PCR2 product (500 μl), NaOAc buffer (50 μl, 3 M, pH 5.2) and ethanol (1.25 ml, -20°C) were mixed and placed at -20°C for 10 min. The PCR2 product was pelleted by centrifugation at 12,000 g for 10 min. The pellet was washed once by 70% v/v ethanol (1 ml, –20°C) after discarding the supernatant. Finally, the aptamer coding strand was purified by dPAGE. The gel band containing the DNA was visualized by the UV shadow method, cut out, and then eluted using elution buffer (500 μl, 200 mM NaCl, 10 mM Tris, 1 mM EDTA, pH 7.5). The DNA was further concentrated by ethanol precipitation as described above. The purified DNA was quantified by UV-Vis absorbance at 260 nm and utilized for the next round of selection. A total of three rounds of magnetic bead-based SELEX was carried out.

Next, the native gel-based selection was employed to further enrich the DNA library. The purified PCR2 product from round 3 above was dissolved with 1× SB (10 μl) and heated at 90°C for 1 min, followed by annealing at room temperature for 10 min. Then, free S1 protein in 1× SB (10 μl) was introduced and incubated at 23°C for 30 min. The DNA-protein complex was separated from unbound DNA by 10% (v/v) native PAGE, which was analyzed by phosphorimaging (Typhoon™ FLA 9500, GE Healthcare, USA). Afterward, the DNA was eluted from the gel with 1× Taq buffer (200 μl) by incubation at 23°C for 20 min. The DNA in the supernatant was collected, followed by the addition of RP1 (10 μl, 10 μM), FP1 (10 μl, 10 μM), Taq DNA polymerase (2 μl, 5 U/μl), and dNTP (20 μl, 2 mM) for PCR1. This was followed by PCR2 using the primer set FP1 and RP2 as described above. Finally, the PCR2 products were purified by dPAGE and utilized for the next round of SELEX. A total of 10 rounds of gel-based SELEX was carried out. Selected DNA libraries were amplified by PCR using primers with sequencing tags and then analyzed using the MiSeq (Illumina) sequencing platform using our previously published protocols (24).

### Phosphorylation of DNA aptamers

DNA aptamers were labeled with γ-[^32^P] ATP at the 5′-end using PNK reactions according to the manufacturer's protocol. Briefly, 2 μl of 1 μM DNA aptamers were mixed with 2 μl γ-[^32^P] ATP, 1 μl of 10× PNK reaction buffer A, 1 μl PNK and 4 μl water. The mixture was incubated at 37°C for 20 min, then purified by 10% dPAGE.

### Dot-blot binding assays

Dot-blot assays were performed using a Whatman Minifold-1 96 well apparatus linked to a vacuum pump. Before experiments, nitrocellulose membranes and nylon membranes were incubated in 1× SB (50 mM HEPES, pH 7.4, 150 mM NaCl, 6 mM KCl, 2.5 mM MgCl_2_, 2.5 mM CaCl_2_, 0.01% Tween-20) for 1 h. The γ-[^32^P] labeled DNA aptamer (4 μl, 10 nM) was dissolved in 196 μl of 1× SB and heated at 90°C for 5 min, then cooled at room temperature for 20 min. The protein partner was dissolved and diluted in the same buffer. 5 μl of the aptamer solution was mixed with 15 μl of the protein solution with different concentrations (0–500 nM). The mixture was incubated at room temperature for 1 h. The dot-blot apparatus was assembled with a nitrocellulose membrane on the top, a nylon membrane in the middle, and a wetted Whatman paper at the bottom. After washing each well with 100 μl of 1× SB, the DNA-protein mixtures were loaded and drained by the vacuum pump (force: 550 mmHg for 8 s). The wells were then washed twice with 100 μl of 1× SB. The radioactivity in the membrane was measured via phosphor storage and a Typhoon 9200 imager (GE Healthcare) and analyzed using Image J software (Molecular Dynamics). The binding between aptamers and pseudotyped or control viruses was performed similarly using the same method except for the viral concentrations (0–900 pM) and incubation time (20 min). The dot blot experiments with saliva samples were also done similarly.

### Experimental details for Figure 2

The dot blot assays were performed as described above. Each binding assay was performed 3 times. The bound fraction (membrane-bound fraction) was quantified and plotted against the concentration of the protein. The *K*_d_ values were derived via curve fitting using Origin 8.0 using the equation: *Y* = *B*_max_*Y*/(*K*_d_ + *X*) (*Y* is the bound fraction of aptamer with protein, *B*_max_ is the maximum bound fraction of aptamer, and *X* is protein concentration).

### Experimental details for Figure 3

The dot blot assays were performed as described for Figure 2. For the specificity test, each protein was used at 50 nM. The binding assay in saliva was performed similarly. Each binding assay was performed 3 times. The *K*_d_ values were obtained using the same methods as described for Figure 2.

### Experimental details for Figures 4, 6 and 7

The dot blot assays were performed and the *K*_d_ values were obtained as described for Figure 2. Each binding assay was performed 3 times.

### Experimental details for Figure 5

The colorimetric assay was conducted on a streptavidin-coated microtiter plate. The wells of the microtiter plate were washed three times with washing buffer (200 μl, 1× SB with 0.1% v/v BSA) after the attachment of each reagent. Briefly, blocking buffer (200 μl, PBST, 10% w/v BSA) was first added to the wells and incubated at 37°C for 1 h to block the wells. Second, biotinylated MSA1 (100 μl, 500 nM) in 1× dilution buffer (1× SB with 2% v/v BSA) was introduced (to bind streptavidin) by shaking at 70 rpm at 22°C for 30 min. Then, different concentrations of the pseudotyped lentivirus of SARS-CoV-2 spiked in 50% (v/v) saliva (diluted with 1× dilution buffer) were added and shaken at 70 rpm at 22°C for 60 min (viral capture by the aptamer on the plate). Next, biotinylated MSA1 (100 μl, 500 nM) in dilution buffer was added again and shaken at 70 rpm at 22°C for 30 min (reporter aptamer binding with the virus). Finally, streptavidin-conjugated HRP (100 μl, 1:1000 dilution) in dilution buffer was added and shaken at 70 rpm at 22°C for 30 min (to bind the reporter aptamer). TMB substrate (100 μl) was then introduced and incubated at 22°C for 20 min. The catalytic reaction was terminated by H_2_SO_4_ (20 μl, 2 M). A plate reader was used to measure the absorbance at 450 nm.

## RESULTS

### Selection of DNA aptamers against SARS-CoV-2 S1 protein (S1 protein)

A library of ∼6 × 10^14^ DNA molecules with 40 nucleotides in the random domain was used for the aptamer selection (the sequences of all synthetic oligonucleotides are provided in Supplementary Table S1). In addition to the 40-nt (nt: nucleotide) random region, we designed the two flanking constant regions such that they would create a stable pairing element (named P1; Supplementary Figure S1A). This was made for two reasons. First, many high-affinity DNA aptamers adopt hairpin structures (25–28), and therefore, this design might populate the library with many aptamer-like structures, which could work to enhance the chance of finding diverse, high-affinity DNA aptamers. Second, engaging the two constant-regions into a predefined pairing element could help prevent them from playing an important role in the recognition of the S1 protein. Ultimately this will simplify the task of establishing secondary structures and minimizing the sizes of selected aptamers.

A combination of a magnetic bead-based (for the first 3 rounds) and EMSA-based (for the subsequent 10 rounds) selection methods were used to derive the S1 protein binding aptamers from the DNA library. For bead-based selection (Supplementary Figure S2A), the histidine tag-containing S1 protein was conjugated onto nickel-nitrilotriacetic acid (Ni-NTA) modified magnetic beads, which were then incubated with the DNA library. The unbound sequences were removed, followed by washes with binding buffer. The bead-bound sequences were eluted, and amplified by PCR to regenerate an enriched pool using a previously published protocol (29,30). For EMSA-based selection (Supplementary Figure S2B), the free S1 protein was first incubated with the DNA pool, followed by separation of the S1-DNA complexes from the unbound DNA using native polyacrylamide gel electrophoresis. After elution from the gel, the bound DNA was amplified by PCR to make a new pool for the next round of selection.

Two PCR steps were conducted for each round, the first with the primer set of FP1 and RP1 and the second with that of FP1 and RP2 (Supplementary Figure S1B). RP2 contained the sequence of RP1 linked to 20 thymidines via a non-amplifiable, 18-atom hexa-ethylene glycol linker (L), and therefore, the non-aptameric strand within the double-stranded DNA product from PCR2 is longer than the aptameric strand by 20 nucleotides. This arrangement facilitated the separation of the aptameric strands by denaturing (8 M urea) gel electrophoresis (dPAGE), a strategy that has been successfully used in many selection experiments conducted in our laboratories (29,30).

The selection pressure was gradually increased by decreasing the library and protein concentrations (Supplementary Table S2). Specifically, for the bead-based selection (rounds 1–3), the concentration of the DNA pool was reduced from 50 μM in round 1, to 2 μM in round 2, and 220 nM in round 3. Similarly, the concentration of the S1 protein was reduced from 3.2 μM in round 1, to 1.6 μM in round 2, and 800 nM in round 3. After switching to the gel-based selection (rounds 4–13), the DNA concentration in round 4 was set at 1 μM, but reduced to 500 nM in round 5, 250 nM in round 6, and 50 nM for the remaining rounds. The protein concentration was used at 3.3 μM in rounds 4–6, then reduced to 1.32 μM in round 6, 660 nM in round 6, and 160 nM for the remaining rounds.

We assessed the original DNA library (Pool 0), as well as the 5th, 7th, 9th, 10th, 11th and 13th pools for binding to the S1 protein by EMSA. The data presented in Supplementary Figure S3 confirmed that our selection was successful. There was a significant jump in the S1 protein binding activity from the 7th pool to the 9th pool. Strong binding activity was also observed for the later pools but the level of binding did not increase significantly (Supplementary Figure S3).

**Figure 1.**
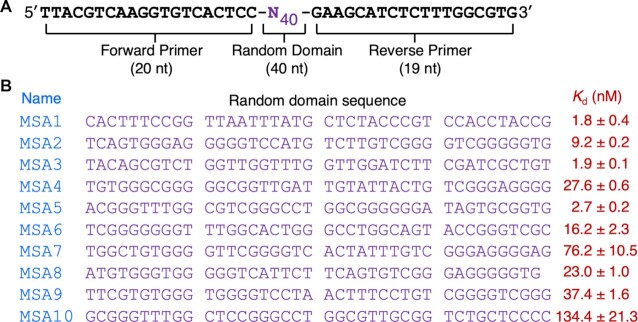
Aptamer selection. (**A**) The sequence of the DNA library used for in vitro selection. (**B**) The top 10 DNA sequences in pool 13 and their binding affinity.

High-throughput sequencing was then conducted with the 13th pool using a previously described protocol (24). Many sequences were discovered; the top 10 ranked sequences are provided in Figure 1B; the top 100 sequences are listed in Supplementary Table S3. The aptamers are named MSAX where MSA stands for Monomeric Spike-binding DNA Aptamer and *X* is the numeral that represents the ranking of the aptamer. For example, MSA1 and MSA5 refer to the top-ranked and 5th-ranked DNA aptamer in the 13th pool.

### Assessment of binding affinity of the top 10 aptamers for S1 protein

**Figure 2.**
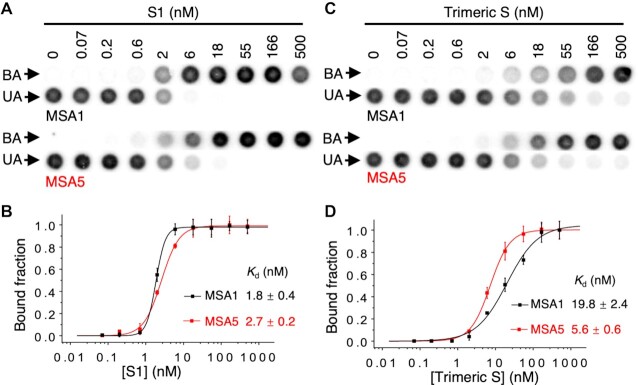
Assessment of binding affinity of MSA1 and MSA5 by dot blot assay. Representative dot blot results showing binding of MSA1 and MSA5 to (**A**) the S1 protein and (**C**) the trimeric S protein of SARS-CoV-2. BA: bound aptamer; UA: unbound aptamer. Binding curves used to derive the *K*_d_ values for MSA1 and MSA5 for (**B**) the S1 protein and (**D**) the trimeric S protein of SARS-CoV-2.

The standard dot blot assay was used to assess the binding affinity of the selected aptamers, a technique that has been widely used to determine the affinity of protein binding aptamers (31–33). Two representative dot blots of MSA1 and MSA5 are provided in Figure 2A. The data was then used to derive the dissociation constant (*K*_d_) for each aptamer (Figure 2B). These analyses were carried for all of the top 10 aptamers and their *K*_d_ values are provided in Figure 1B. Using this assay, all the aptamers showed binding to the S1 protein, although their *K*_d_ values varied substantially: MSA1, MSA3 and MSA5 represent the three highest affinity aptamers, with *K*_d_ values of 1.8, 1.9 and 2.7 nM respectively. These *K*_d_ values are better than other reported RBD-binding or S1 protein-binding DNA aptamers (Supplementary Table S4), indicating our aptamers have higher affinities, though we note that a recently reported bivalent RBD-binding circular DNA aptamer has a reported *K*_d_ of 0.13 nM (20). MSA7 and MSA10 are the two worst aptamers, with *K*_d_ values of approximately 100 nM. The other top 5 aptamers have *K*_d_ values in the range from ∼10–40 nM, which are similar to most of the previously reported aptamers.

### Assessment of binding affinity of MSA1 and MSA5 for the full spike protein

Our aptamers were selected to bind the S1 subunit of the wild-type SARS-CoV-2 spike protein. However, the full spike protein is a trimer, with each subunit consisting of a heterodimer containing a S1 and S2 domain covalently linked to each other (34), and thus we also tested the binding of aptamers to the trimeric spike proteins of SARS-CoV-2. As the trimeric spike proteins are densely glycosylated proteins (35), it is functionally important that the aptamers be able to recognize the trimeric S complex.

For these studies we decided to move forward with the MSA1 and MSA5 aptamers, two of the best aptamers from our selection in terms of affinity for the S1 protein. MSA1 was chosen both for its top ranking and highest affinity; MSA5 was picked for its high affinity and its G-rich sequence property, which is a common motif for many reported DNA aptamers (36). The dot blot assays shown in Figure 2C demonstrate that both MSA1 and MSA5 still exhibit strong binding to the trimeric S-protein complex, with *K*_d_ values of 19.8 and 5.6 nM, respectively (Figure 2D). The decrease in affinity may be due to the structural differences between the monomeric S1 and the trimeric S complex as well as the glycosylation of the trimeric S complex.

### Assessment of binding affinity of MSA1 and MSA5 for the RBD of the spike protein

We were curious to determine if the featured aptamers MSA1 and MSA5 would also bind to the RBD of the spike protein, given they were selected using the RBD-containing S1 protein. The dot blot assays shown in Supplementary Figure S4 indicate that MSA1 and MSA5 still recognize the RBD with high affinity, as reflected by the *K*_d_ values (3.1 and 4.0 nM, respectively), which are only slightly higher than their *K*_d_ values for the S1 protein (1.8 and 2.7 nM, respectively). Work is underway to examine other aptamers from our selection that recognize non-RBD epitopes on the spike protein.

### Selectivity assessment of MSA1 and MSA5

We next tested the specificity of MSA1 and MSA5 by evaluating their binding to the following four proteins: Bovine serum albumin (BSA), human α-thrombin (Tb), human immunoglobulin G (IgG) and RNase H2 of *Clostridium difficile* (CD-RNase H2). BSA is commonly used as a control protein to test the specificity of aptamers. IgG is an important antibody present in human blood and saliva. Human α-thrombin was chosen as a control protein because it has been widely used in aptamer studies due to the existence of a high-affinity DNA aptamer that specifically recognizes this human protein (37,38). CD-RNase H2 was chosen to represent a nucleic acid binding protein so as to assess potential non-specific binding to nucleases and other DNA-binding proteins potentially present in blood or saliva.

**Figure 3.**
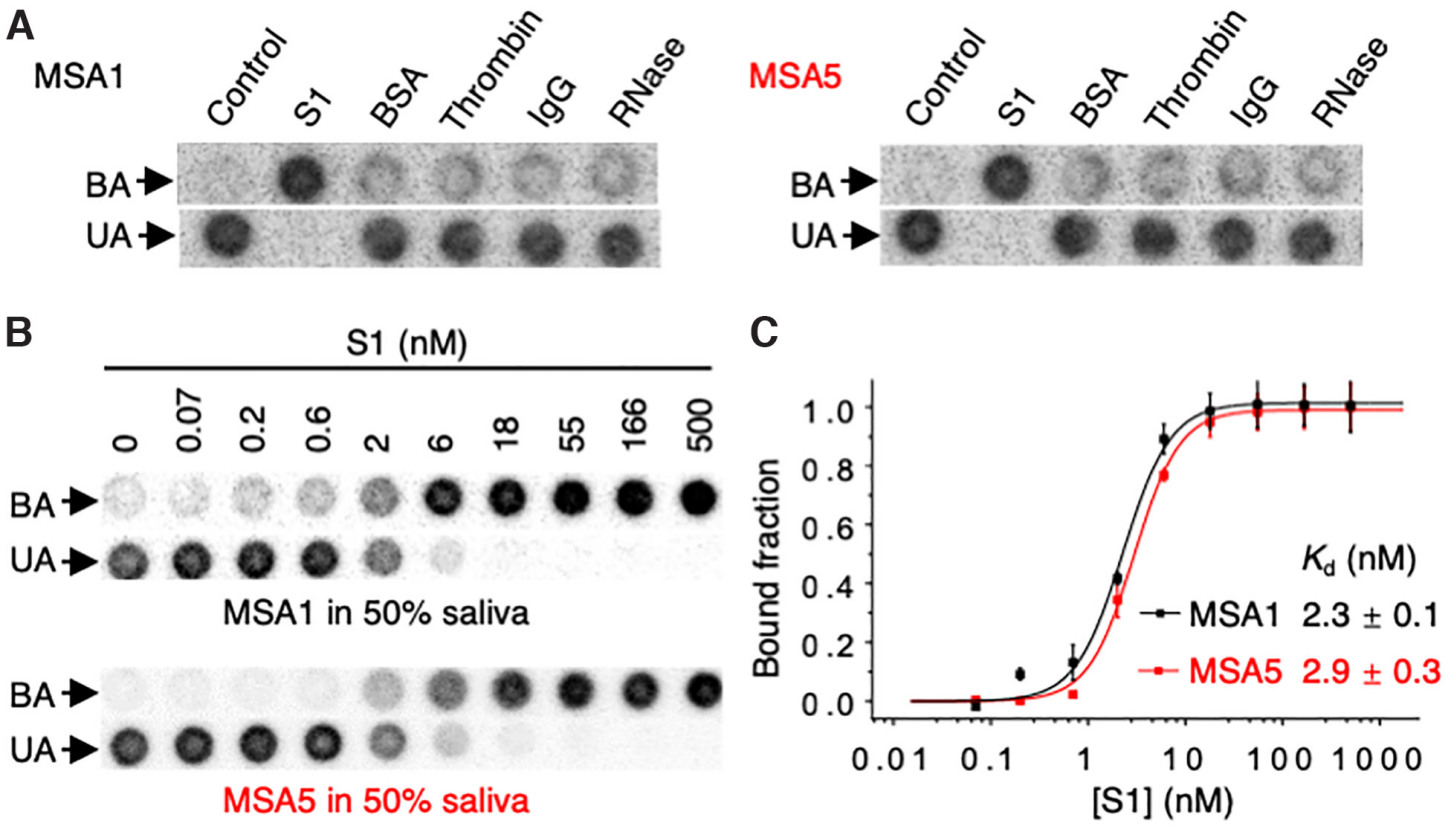
(**A**) Dot blot results of MSA1 and MSA5 for binding to the S1 protein of SARS-CoV-2 and control proteins. (**B**) Dot blot results and (**C**) affinity (*K*_d_) of MSA1 and MSA5 for binding to the S1 protein in the 50% pooled human saliva. For dot blots, BA: bound aptamer; UA: unbound aptamer.

Figure 3A shows dot blot assay data for both MSA1 and MSA5 binding to these proteins, along with the use of the S1 protein as the positive control and binding buffer as the no-protein control. The data clearly indicates that none of the four control proteins showed detectable binding to either aptamer, indicating that both MSA1 and MSA5 recognize S1 protein highly specifically and that common human proteins in biological fluids and proteins with intrinsic nucleic acid binding properties do not interact with the aptamers.

Using the dot blot assay, we also tested the binding of MSA1 and MSA5 with an additional four relevant proteins, including the RBD and the spike protein of SARS-CoV-1, the RBD of Middle East Respiratory Syndrome (MERS) and the RBD of Coronavirus HCoV-229E, a seasonal human coronavirus; the data are provided in Supplementary Figure S5. MSA1 shows weak binding to the spike protein of SARS-CoV-1 and the RBD of MERS (Supplementary Figure S5A), whereas MSA5 did not recognize any of these control proteins (Supplementary Figure S5B). However, the binding affinity of MSA1 for the spike protein of SARS-CoV-1 and the RBD of MERS was very poor, with *K*_d_ values greater than 150 nM and 100 nM, respectively (Supplementary Figure S6). Given that both SARS-CoV-1 and MERS are no longer in circulation and that the aptamer does not recognize HCoV-229E, it is clear that MSA1 and MSA5 show sufficient selectivity for SARS-CoV-2.

### Binding of S1 protein by MSA1 and MSA5 in human saliva

One potential application of these aptamers is to detect SARS-CoV-2 in saliva specimens, which could allow self-sample collection to make testing for SARS-CoV-2 easier. To examine their functionality in human saliva, MSA1 and MSA5 were tested for binding to the S1 protein spiked into 50% pooled human saliva (diluted with binding buffer) using dot blot assays (Figure 3B). Both aptamers performed well in 50% saliva, producing similar *K*_d_ values in saliva (Figure 3C) relative to binding buffer (Figure 2B).

Human saliva represents a very complex biological fluid, which contains a variety of proteins including digestive enzymes and nucleic acid binding proteins, as well as polysaccharides, mucus and electrolytes (39,40). The full functionality of MSA1 and MSA5 towards the S1 protein in saliva further confirms the high selectivity of the aptamers and points to the potential utility of using these aptamers for SARS-CoV-2 diagnostics.

### Binding of the spike protein of the UK variant by MSA1 and MSA5

The spike protein of coronaviruses, particularly their receptor binding domains (RBDs), are known to mutate frequently (41). Several variants of concern of SARS-CoV-2 have emerged, among which is the B.1.1.7 variant that emerged in the United Kingdom (UK) and was first reported in September 2020 (42). This variant is also known as 501Y.V1 and has a N501Y mutation in the RBD. The B.1.1.7 variant has now spread globally and in some countries, such as Canada, it has become the dominant SARS-CoV-2 virus in circulation.

**Figure 4.**
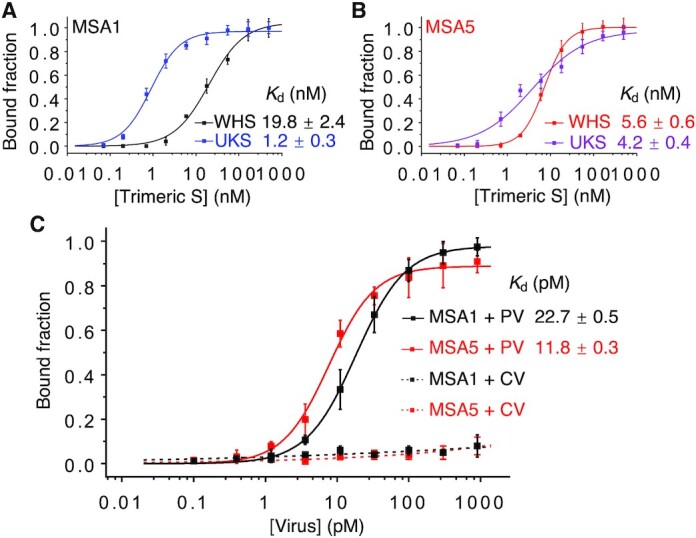
Affinity (*K*_d_) analysis of binding of (**A**) MSA1 and (**B**) MSA5 to the trimeric S proteins of the original Wuhan variant (WHS) and the B.1.1.7 variant (UKS). (**C**) Affinity (*K*_d_) analysis of binding of MSA1 and MSA5 to a pseudotyped lentivirus (PV) that was engineered to display the S-protein of SARS-CoV-2 and the same lentivirus that lacks the S-protein (CV).

To determine if MSA1 and MSA5 could still recognize the mutated spike protein of the B.1.1.7 variant, we performed dot blot assays to compare the binding of MSA1 (Supplementary Figure S7A) and MSA5 (Supplementary Figure S7B) to the trimeric S protein of both the wild-type Wuhan virus (WHS) and B.1.1.7 variant (UKS). Interestingly, as seen in Figure 4A, the MSA1 aptamer showed significantly higher affinity for the UKS (*K*_d_ of 1.2 nM) relative to the WHS (*K*_d_ of 19.8 nM). MSA5 exhibited nearly identical *K*_d_ values for the trimeric S protein of both the wild-type and B.1.1.7 variants (Figure 4B). While the binding of MSA5 was not affected by the N501Y mutation, the MSA1 aptamer bound with 16-fold higher affinity to the UK variant with the N501Y mutation, suggesting that this mutation is likely within the binding domain for MSA1. Intriguingly, this observation also shows that it is possible for an aptamer to show stronger binding to a mutant, even though it was not the target used for selection.

### Binding of a pseudotyped SARS-CoV-2 lentivirus by MSA1 and MSA5

Next, we tested MSA1 and MSA5 for binding to a pseudotyped SARS-CoV-2 lentivirus. Specifically we used a pseudotyped lentivirus (PV) that was engineered to display the full trimeric S-protein of SARS-CoV-2 within the viral envelope (23,43,44). The surface of this virus resembles that of SARS-CoV-2; it can enter human cells but cannot replicate itself, allowing for its use in biosafety-level-2 labs as a model virus (23). The same lentivirus that lacks the S-protein was used as a control virus (CV) for this experiment.

Dot blot assays were performed with both the PV (Supplementary Figure S8A) and the CV (Supplementary Supplementary Figure S8B) for MSA1 and MSA5, and the binding data is presented in Figure 4C (see the experimental section for a description of how the viral concentration was determined). Both MSA1 and MSA5 were found to recognize the PV (Supplementary Figure S8A) but not the CV (Supplementary Figure S8B). The *K*_d_ values for PV were determined to be 22.7 pM and 11.8 pM, respectively, for MSA1 and MSA5 (Figure 4C). The increased affinity in comparison to the purified S1 protein can be explained by the fact that each viral particle carries many copies of the S-protein. Although the copy number of the spike protein on the surface of the SARS-CoV-2 viruses has been reported to be ∼30 (45,46), the copy number on the viral particles of the PV has not been reported. However, if we assume each PV virus carries 100 copies of the S-protein, the protein-equivalent *K*_d_ values for MSA1 and MSA5 are estimated to be 2.3 and 1.2 nM, which are close to the *K*_d_ values for the S1 protein (1.8 and 2.7 nM, respectively). Importantly, both MSA1 and MSA5 can recognize fully functional spike proteins of SARS-CoV-2, even though they were selected using the purified S1 protein.

### Binding of MSA1 and MSA5 to heat-treated S1 protein and the pseudotyped SARS-CoV-2 virus

Working with either SARS-CoV-2 viruses or patient clinical samples containing the SARS-CoV-2 virus poses a high risk of acquiring infection for health care providers and research personnel. One simple way to deactivate the virus is to use heat. For example, many viruses can be simply deactivated at 60°C for 30 min, 65°C for 15 min or 80°C for 1 min (47). It has been reported that heat treatment for 5 min at temperatures above 65°C can completely deactivate SARS-CoV-2 viruses (48). Based on these observations, heat deactivation of the clinical samples before testing can effectively avoid infection during the assay. However, it is also possible that heat deactivation can significantly affect the integrity of the spike protein of SARS-CoV-2, resulting in the loss of antigenicity of the virus. For example, heat inactivation for immunoanalysis of antibodies to SARS-CoV-2 is not recommended due to the possibility of false-negative results when the serum samples are pre-inactivated at 56°C for 30 minutes (49).

Aptamer binding to both the S1 protein and PV was assessed after each of these samples was heated at 65°C for 60 min. Surprisingly, both MSA1 and MSA5 maintained nearly full binding activity to both the S1 protein and PV following heat treatment (Supplementary Figure S9). This observation shows that the spike protein is thermally stable and implies that both MSA1 and MSA5 are compatible with a safe test protocol for SARS-CoV-2 detection that involves a viral deactivation step at 65°C.

### A colorimetric assay for viral detection in Saliva using MSA1

**Figure 5.**
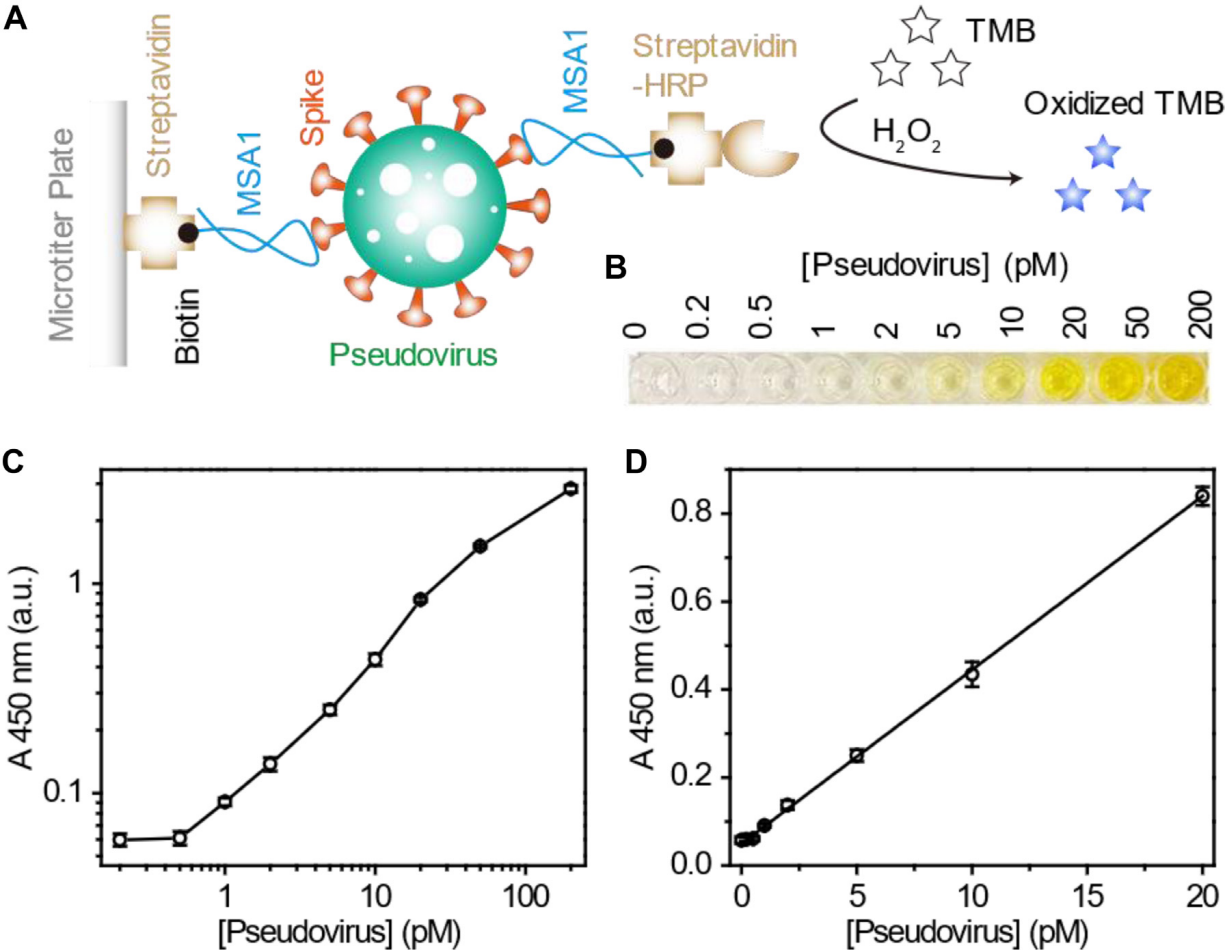
Proof of concept for a colorimetric assay to detect the pseudotyped lentivirus of SARS-CoV-2 in 50% saliva via a sandwich assay using MSA1. (**A**) The assay principle. (**B**) Photograph of the colorimetric test. (**C**) Absorbance at 450 nm of the reaction solutions. (**D**) The linear response curve in the range of 0.5–20 pM pseudovirus.

We next examined the possibility of using our aptamers to develop a colorimetric assay to detect the PV in 50% pooled saliva. The MSA1 aptamer was used for this demonstration. Because each viral particle carries multiple spike proteins on its surface, we designed a sandwich assay that uses two identical biotinylated MSA1 aptamers to bind a single viral particle (Figure 5A). The first aptamer was biotinylated at the 3′ end and immobilized onto a 96-well microtiter plate coated with streptavidin. The second biotinylated MSA1 aptamer was tagged with horseradish peroxidase (HRP) conjugated to streptavidin. Formation of the biotin-streptavidin conjugate prior to the assay avoids the potential for interference by endogenous biotin in clinical samples, which has been reported in some ELISA assays (50). The presence of the PV led to immobilization of HRP onto the plate, which oxidized 3,3′,5,5′-tetramethylbenzidine (TMB) in the presence of H_2_O_2_. After quenching the reaction with H_2_SO_4_, the blue-colored oxidized TMB turned yellow and was measured at 450 nm.

As shown in Figure 5B, the yellow color of the reaction mixture intensified with increasing concentrations of the PV. The visual limit of detection (LOD) was 1 pM. The absorbance at 450 nM versus the concentration of the PV is provided in Figure 5C, which showed an increase in *A*_450_ with increasing viral concentration. A linear response curve in the range of 0.5–20 pM was observed (Figure 5D). The LOD, defined by 3 times the standard deviation of the blank samples, was determined to be 400 fM, which corresponds to 2.4 × 10^8^ virus particles per ml. This viral concentration, equivalent to a C_t_ value of ∼16, is in the upper range of viral particle concentration for an infected patient (normal range for infected patients is 10^3^ to 10^11^ viral particles per ml (51). The LOD could be improved by either performing a pre-concentration step or by reformatting the test to produce a more sensitive readout, such as fluorescence. We are currently working on more sensitive assay formats that do not require laboratory instrumentation, which will be reported in a future manuscript.

### Secondary structure analysis of MSA1, MSA5 and MSA3

We have shown above that our aptamer selection experiments have resulted in the isolation of three aptamers, MSA1, MSA3 and MSA5, that recognize the S1 protein with single-digit nanomolar *K*_d_ values (Figure 1). As noted above, these aptamers were isolated from a DNA library pre-engineered to contain a pairing element to confine the two constant-sequence domains (Supplementary Figure S1), and hence we examined the secondary structures of these aptamers to determine if this structural motif was present in each of the aptamers and made attempts to minimize their sizes via the examination of truncation mutants so that shortened aptamer constructs with defined secondary structures could be established for future applications.

**Figure 6.**
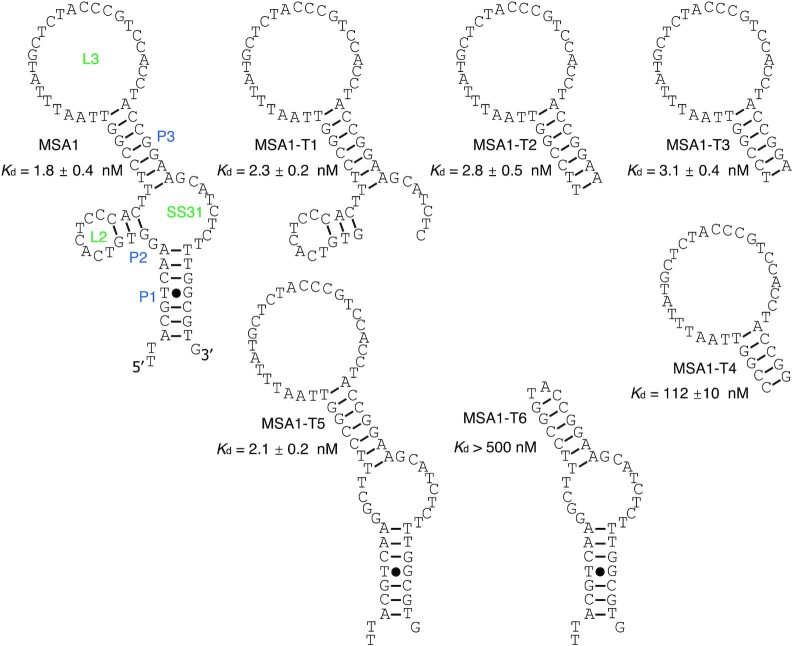
The predicted secondary structure of MSA1 and the binding affinity of its truncation mutants.

The predicted structure of MSA1 using the mfold program is provided in Figure 6. In addition to the pre-engineered P1 element, MSA1 was predicted to contain 2 additional pairing elements (3-bp P2 and 7-bp P3; P: pairing element; bp: base-pair) and 3 unpaired elements (7-nt L2, 25-nt L3, 8-nt SS31; nt: nucleotide; L: loop; SS: single-stranded element; SS31: the single-stranded element linking P3 to P1).

Six truncation mutants of MSA1, named MSA1-T1 to MSA1-T6, were tested for binding to the S1 protein using the dot blot assay. The results indicated that P3-L3 elements are important for target recognition as the truncation mutant MSA1-T2, in which P3 and L3 are kept but all of the other elements are eliminated, retained full binding activity. The length of P3 can be reduced from 7-bp to 6-bp, as the resultant mutant MSA1-T3 exhibited similar binding affinity. Further reduction of P3 to 5-bp resulted in substantial loss of activity (increase of *K*_d_ by 36-fold comparing MSA1-T3 and MSA1-T4). The elimination of L3, however, abolished the binding ability of the aptamer (MSA1-T6; *K*_d_ > 500 nM).

**Figure 7.**
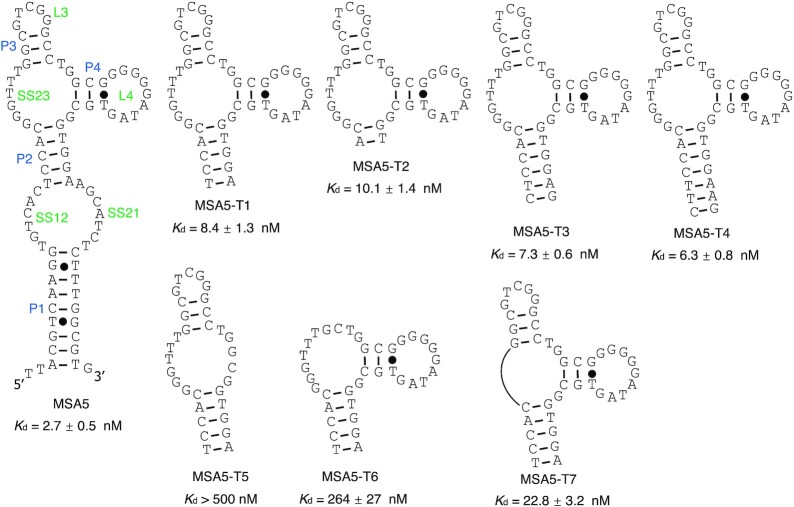
The predicted secondary structure of MSA5 and the binding activity of its truncation mutants.

The predicted structure of MSA5 is provided in Figure 7. The overall structure contains 4 pairing elements (9-bp P1, 5-bp P2, 3-bp P3 and 3-bp P4) and 5 unpaired elements (6-nt SS12, 7-nt SS21, 6-nt SS23, 5-nt L3, 9-nt L4). Four truncation mutants of MSA5 were examined by the dot blot assay (named MSA5-T1 to MSA5-T4, respectively). Removing P1, SS12 and SS21 together (loss of 34 nucleotides) led to a significantly truncated mutant, MSA5-T1, that still exhibited strong binding activity (*K*_d_ of 8.4 nM; a 3.1-fold increase over that of the full-length aptamer). Further reduction of P2 in MSA5-T1 from 5-bp to 2-bp (MSA5-T2; *K*_d_ of 10.1 nM; 3.7-fold increase) or increasing the length of P2 to 6-bp (MSA5-T3; *K*_d_ of 7.3 nM; 2.7-fold increase) and 7-bp (MSA5-T4; *K*_d_ of 6.3 nM; 2.3-fold increase) also led to mutants with similar binding affinity to that of MSA5-T1, although mutants with the stronger P2 elements exhibited better binding affinity. Truncation of P4-L4 (MSA5-T5; *K*_d_ > 500 nM;) or P3-L3 (MSA5-T6; *K*_d_ of 264 nM) drastically affected the binding activity. Removal of SS23 (MSA5-T7; *K*_d_ of 22.8 nM; 8.4-fold increase) also affected the binding affinity substantially but did not inactivate the binding. These results indicate that the P2, SS23, P3, L3, P4 and L4 elements play important roles in the target recognition and the other structural elements can be eliminated.

The predicted structure of MSA3 is provided in Supplementary Figure S10. We examined four truncation mutants (named MSA3-T1 to MSA5-T4, respectively) by the dot blot assay. The results also show that MSA3 also uses a hairpin structure to support its binding. Based on the above analysis, it is clear that all of the best three aptamers in terms of binding affinity adopt a simple hairpin structure to achieve the recognition of the S1 protein (Supplementary Figure S11), as illustrated by their minimized truncation mutants MSA1-T3 (*K*_d_ of 3.1 nM; 37 nt), MSA3-T2 (*K*_d_ of 1.9 nM; 39 nt), MSA5-T4 (*K*_d_ of 6.3 nM; 49 nt).

## DISCUSSION

In summary, we report an effort to select DNA aptamers from a pre-structured synthetic DNA library using the S1 subunit of the spike protein of wild-type SARS-CoV-2 as the binding target, which resulted in the isolation of many different aptamer sequences. Through the examination of the binding affinity and selectivity of the top 10 sequences, we confirmed that the isolated sequences represent high-quality aptamers, with the best three aptamers exhibiting *K*_d_ values of 1 - 3 nM for the S1 subunit. The top-ranking DNA aptamer, MSA1, has a *K*_d_ of 1.8 nM; for comparison, the best *K*_d_ value for binding to the S1 protein by previously reported aptamers is 13 nM (19), which would translate to a 7-fold poorer limit of detection. We also performed dot blot assays with two previously reported aptamers, CoV2-RBD-1 (17) and Apt-S-268s (21), to assess binding to the spike protein of SARS-CoV-2 and determined their *K*_d_ values (Supplementary Figure S12). Our experiment confirmed the expected affinity of the CoV2-RBD-1 aptamer (Supplementary Figure S12A), with a *K*_d_ value of 37.4 nM (Supplementary Figure S12B), though this is significantly poorer than MSA1 and MSA5. Apt-S-268s, however, showed very weak binding to the spike protein (Supplementary Figure S12A), with an estimated *K*_d_ value greater than 200 nM (Supplementary Figure S12B).

An important point that is worth noting is our approach of structuring a DNA library with a strong pairing element, which may be a key factor that is responsible for rapidly discovering numerous high-affinity aptamers. We initially tested the top 10 aptamer candidates and found that all of them bind the S1 protein with excellent affinity (Figure 1). We further examined the binding affinity of three other aptamers, including MSA11 and two low-ranked aptamers, MSA50 and MSA439. We found that all three aptamers still showed excellent affinity, with *K*_d_ values of 30.1, 10.2 and 36.9 nM, respectively (Supplementary Table S3). Therefore, we believe that our structured library approach, in which the two constant-sequence regions are engaged into a strong duplex, represents an attractive option for aptamer discovery. Of note, the structured library approach has been used for the successful discovery of diverse kinase ribozymes (52) and RNA-cleaving DNAzymes (53). The structured library approach has also been used by Davis and Szostak for the creation of GTP-binding RNA aptamers (54). Specifically, they inserted a short stem-loop in the middle of the random region and combined this library with a non-structured pool to derive GTP-binding aptamers. In this case, the structured library outcompeted the non-structured sequences as all selected aptamers contained the hairpin insert.

The second advantage of using the structured DNA library for aptamer selection is the reduced workload for establishing secondary structures and obtaining minimized sequences of isolated aptamers. In our approach, we placed the two constant regions that flank the random domain into a stable pairing element. In essence, we structured the DNA library into a hairpin structure, with the intention of preventing them from playing an important role in the recognition of the S1 protein so that they could be easily removed. The results from the secondary structure prediction and sequence truncation analysis applied to the three best aptamers identified from our selection, MSA1, MSA3 and MSA5, confirm that this approach could indeed simplify the task of establishing secondary structures and minimizing the sizes of selected aptamers.

Several other DNA aptamers have been selected to bind the receptor binding domain (RBD) (17,20), the S1 protein (21), trimerized S1 protein (19), and virus mimics (18). We performed a BLAST (55) comparison of our top 10 aptamers with these aptamers and found our aptamers to be distinct from all other published aptamers surveyed (Supplementary Figure S13A). The highest scoring sequence similarity was observed between MSA2 and a published aptamer named Apt-S-79s as well as between MSA10 and another published aptamer CoV-2; however, alignment of the sequences occurs in the G-rich regions of both aptamer pairs (Supplementary Figure S13B) and may not be indicative of a functional element as G-rich sequences are commonly found in a wide range of aptamer selections (36).

Our DNA aptamers were isolated to bind the purified S1 subunit of the spike protein of SARS-CoV-2. However, our featured aptamers, MSA1 and MSA5, could also recognize the full spike protein of SARS-CoV-2 and the pseudotyped lentivirus that expresses the full spike protein of SARS-CoV-2. It is known that the S1 subunit is well folded into a stable structure that closely resembles the full trimeric spike protein (34). The observation of the equivalent binding of MSA1 and MSA5 to the S1 protein, the trimeric spike protein and the pseudotyped virus supports the structural and functional similarity of the S1 protein to the functional spike on the virus.

Interestingly, we observed that both MSA1 and MSA5 were still able to bind the S1 protein and the pseudotyped virus after they were treated at 65°C for 60 minutes. This suggests that our aptamers appear to recognize an epitope (or epitopes) of the spike protein that is heat resistant. Given that treating the SARS-CoV-2 virus at 65°C for as short as 5 minutes can completely deactivate it (48), our aptamers can be incorporated into a safe diagnostic method where heat deactivation of clinical samples can be used to avoid infection during the assay, which may be of importance if used in congregate settings for screening multiple samples.

Furthermore, we found that both MSA1 and MSA5 exhibited similar binding affinity to the trimeric S protein of both the wild-type Wuhan virus and the B.1.1.7 variant, even though these aptamers were selected using the S1 subunit of the spike protein from the wild-type virus. At present, we do not know the precise binding site of the aptamers on the spike protein, though this observation points to the possibility that the binding site may not be at or near the N501Y mutation in the RBD and/or the conformation of the binding site is not significantly affected by N501Y mutation.

We also found that the aptamers maintained full binding activity with the S1 protein spiked into 50% human saliva, which is an easily accessed sample type that could support home-testing or rapid testing in congregate settings. Based on this finding, we developed a simple colorimetric sandwich assay using the MSA1 aptamer as an MRE and showed that it was capable of detecting pseudotyped lentivirus in 50% saliva with a limit of detection of 400 fM. This proof-of-concept experiment confirmed the potential of these aptamers as diagnostic tools for COVID-19 detection in an easily accessed patient sample. As an on-going effort, we are actively pursuing the use of aptamers to develop rapid antigen tests for the diagnosis of COVID-19 in clinical settings.

## Supplementary Material

gkab574_Supplemental_File
