# Supplementary material for: Diverse high-affinity DNA aptamers for wild-type and B.1.1.7 SARS-CoV-2 spike proteins from a pre-structured DNA library
Source: Nucleic Acids Res. 2021 Jul 7;49(13):7267–79. doi: 10.1093/nar/gkab574 (PMC8287928; doi:10.1093/nar/gkab574)
Supplement: gkab574_Supplemental_File [file gkab574_supplemental_file.pdf]

Table S1. All the synthetic oligonucleotides used in this study. Sequences are written 5′-3′. Abbreviations include: N_{40}: 40-nucleotide random region; L non-amplifiable linker.

**Selection**		
DNA library (79 nt)		TTACGTCAAG GTGTCACTCC-$\mathbf{N_{40}}$-GAAGCATCTC TTTGGCGTG
Forward primer FP1 (20 nt)		TTACGTCAAG GTGTCACTCC
Reverse primer RP1 (19 nt)		CACGCCAAAG AGATGCTTC
Reverse primer RP2 (39 nt)		TTTTTTTTTT TTTTTTTTTT-L-CACGCCAAA GAGAT GCTTC
**Aptamers**		
**Name**	**Size (nt)**	
MSA1	79	TTACGTCAAG GTGTCACTCC CACTTTCCGG TTAATTTATG CTCTACCCGT CCACCTACCG GAAGCATCTC TTTGGCGTG
MSA2	79	TTACGTCAAG GTGTCACTCC TCAGTGGGAG GGGGTCCATG TCTTGTCGGG GTCGGGGGTG GAAGCATCTC TTTGGCGTG
MSA3	79	TTACGTCAAG GTGTCACTCC TACAGCGTCT GGTTGGTTTG GTTGGATCTT CGATCGCTGT GAAGCATCTC TTTGGCGTG
MSA4	79	TTACGTCAAG GTGTCACTCC TGTGGGCGGG GGCGGTTGAT TGTATTACTG TCGGGAGGGG GAAGCATCTC TTTGGCGTG
MSA5	79	TTACGTCAAG GTGTCACTCC ACGGGTTTGG CGTCGGGCCT GGCGGGGGGA TAGTGCGGTG GAAGCATCTC TTTGGCGTG
MSA6	79	TTACGTCAAG GTGTCACTCC TCGGGGGGGT TTGGCACTGG GCCTGGCAGT ACCGGGTCGC GAAGCATCTC TTTGGCGTG
MSA7	79	TTACGTCAAG GTGTCACTCC TGGCTGTGGG GTTCGGGGTC ACTATTTGTC GGGAGGGGAG GAAGCATCTC TTTGGCGTG
MSA8	78	TTACGTCAAG GTGTCACTCC ATGTGGGTGG GGGTCATTCT TCAGTGTCGG GAGGGGGTGG AAGCATCTCT TTGGCGTG
MSA9	79	TTACGTCAAG GTGTCACTCC TTCGTGTGGG TGGGGTCCTA ACTTTCCTGT CGGGGTCGGG GAAGCATCTC TTTGGCGTG
MSA10	79	TTACGTCAAG GTGTCACTCC GCGGGTTTGG CTCCGGGCCT GGCGTTGCGG TCTGCTCCCC GAAGCATCTC TTTGGCGTG
MSA1-T1	60	GTGTCACTCC CACTTTCCGG TTAATTTATG CTCTACCCGT CCACCTACCG GAAGCATCTC
MSA1-T2	39	TTCCGGTTAA TTTATGCTCT ACCCGTCCAC CTACCGGAA
MSA1-T3	37	TCCGGTTAAT TTATGCTCTA CCCGTCCACC TACCGGA
MSA1-T4	35	CCGGTTAATT TATGCTCTAC CCGTCCACCT ACCGG
MSA1-T5	68	TTACGTCAAG GCTTTCCGGT TAATTTATGC TCTACCCGTC CACCTACCGG AAGCATCTCT TTGGCGTG
MSA1-T6	43	TTACGTCAAG GCTTTCCGGT ACCGGAAGCA TCTCTTTGGC GTG
MSA3-T1	49	CTCCTACAGC GTCTGGTTGG TTTGGTTGGA TCTTCGATCG CTGTGAAGC
MSA3-T2	39	ACAGCGTCTG GTTGGTTTGG TTGGATCTTC GATCGCTGT
MSA3-T3	34	ACAGCGTCTG GTTGGTTTGG TTGGATCTCG CTGT
MSA3-T4	21	TCTGGTTGGT TTGGTTGGAT C
MSA5-T1	45	TCCACGGGTT TGGCGTCGGG CCTGGCGGGG GGATAGTGCG GTGGA
MSA5-T2	39	ACGGGTTTGG CGTCGGGCCT GGCGGGGGGA TAGTGCGGT
MSA5-T3	47	CTCCACGGGT TTGGCGTCGG GCCTGGCGGG GGGATAGTGC GGTGGAG
MSA5-T4	49	CTTCCACGGG TTTGGCGTCG GGCCTGGCGG GGGGATAGTG CGGTGGAAG
MSA5-T5	32	TCCACGGGTT TGGCGTCGGG CCTGGCGGTG GA
MSA5-T6	36	TCCACGGGTT TGCTGGCGGG GGGATAGTGC GGTGGA
MSA5-T7	39	TCCACGGCGT CGGGCCTGGC GGGGGGATAG TGCGGTGGA
Bio-MSA1	84	B-TTTTTTTACG TCAAGGTGTC ACTCCCACTT TCCGGTTAAT TTATGCTCTA CCCGTCCACC TACCGGAAGC ATCTCTTTGG CGTG

Table S2. Concentrations of DNA and protein used during SELEX.

Selection methods	SELEX rounds	Reaction volume (μL)	[Library] (nM)	[S1] (nM)
Bead-based	1	20	50000	3250
	2	40	2000	1600
	3	80	220	800
EMSA-based	4	20	1000	3300
	5	20	500	3300
	6	20	250	3300
	7	20	50	1320
	8	20	50	660
	9	20	50	160
	10	20	50	160
	11	20	50	160
	12	20	50	160
	13	20	50	160

Table S3. DNA sequences in pool 13 ranked by their percentage.

Rank in pool 13	Sequences (5'→3')[a]	% in pool 13	K_d (nM) [c]
1	CACTTTCCGGTTAATTTATGCTCTACCCGTCCACCTACCG	18.769	1.8 ± 0.4
2	TCAGTGGGAGGGGGTCCATGTCTTGTCGGGGTCGGGGGTG	10.335	9.2 ± 0.2
3	TACAGCGTCTGGTTGGTTTGGTTGGATCTTCGATCGCTGT	6.277	1.9 ± 0.1
4	TGTGGGCGGGGGCGGTTGATTGTATTACTGTCGGGAGGGG	5.904	27.6 ± 0.6
5	ACGGGTTTGGCGTCGGGCCTGGCGGGGGGATAGTGCGGTG	4.699	2.7 ± 0.2
6	TCGGGGGGGTTTGGCACTGGGCCTGGCAGTACCGGGTCGC	4.427	16.2 ± 2.3
7	TGGCTGTGGGGTTCGGGGTCACTATTTGTCGGGAGGGGAG	2.739	76.2 ± 10.5
8	ATGTGGGTGGGGGTCATTCTTCAGTGTCGGGAGGGGGTG	1.923	23.0 ± 1.0
9	TTCGTGTGGGTGGGGTCCTAACTTTCCTGTCGGGGTCGGG	1.915	37.4 ± 1.6
10	GCGGGTTTGGCTCCGGGCCTGGCGTTGCGGTCTGCTCCCC	1.660	134.4 ± 21.3
11	TGTGGGCGGGGGTGTACTGCATTGTCGGGGTAGGGGGATG	1.660	30.1 ± 0.5
12	TGCGGGCGGGGGTGGTTACGCTCTCATCTGTCGGGTGGGG	1.472	
13	TGTGGGCGGGGGATCTAGTGTCTGTCGGGGCCGGGGGGTG	1.052	
14	TCGTGTGGGTGGGGTTGCTTTTGCTTTCTTGTCGGGCGGG	0.803	
15	TCTGGTGGGGTGTGGGGTAGGATTGTCGGGAGGGGAGCAT	0.778	
16	ATGCGGGCGGGGGTCTGATCAGTCTGTCGGGAGGGGGTCG	0.776	
17	TGTGGGTGGGGTTTGTCTGGTTTATTTGTTGTCGGGCGGG	0.757	
18	TGTTGTGGGGGTTACTGGGGGTCATGGCTGTCGGGCGGGG	0.678	
19	TCTTGTGGGACGGGGGATATCTCTGTCGGGGACGGGGGTG	0.625	
20	TCGGTGTGGGTGGGGCTTCATATGTCTGTCGGGGTCGGGG	0.601	
21	TGTGGGTGGGGGTCGATATCCTTGTCGGGGCAGGGGGAG	0.567	
22	AGGTGGGTGGGGTTGTTTGTTGTTTTTATTGTCGGGTGGG	0.501	
23	ACGTGGGCGGGGGAGAAGGTTTGTCGGGGATCGGGGGTAG	0.500	
24	CACTGGGTTTGGCTCTGGGCCTGGCATGTGACCCTGTTCT	0.494	
25	TGCGTGTGGGGTTTGGGGCCATGAATTGTCGGGAGGGGAG	0.488	
26	AGCACGTGGTCATACTGTGCCTGGGTTTTTTGCCCAACGT	0.484	
27	CTCGTGTGGGGTCGTCGGGGTGAGAGTGTCGGGCGGGGTG	0.468	
28	TCACACTGTGGGCGGGGCTAAGGTTTTGTCGGGGCGTGGG	0.428	
29	TGTGGGTGGGGGCGGAGGTTATCAGTCTGTCGGGAGGGGG	0.415	
30	CTCGTGGGCGGGGTTTTATTTTCGTTCTGTCGGGGTTGGG	0.414	
31	AGCACGTGGTCATACTGTGCCTGGGTACTCCGCCCAACGT	0.389	
32	TGCGGGTGGGGGTCTAGTTTGGATCTGTCGGGAGGGGGTG	0.372	
33	TCAGCGGGTGCTTGGTTGGTCTGGTTTGGTGTCCCCGGGA	0.369	
34	TCTGTGTGGGACGGGGGCCGAACTGTCGGGGTAGGGGGTG	0.346	
35	TGGTGGGCGGGTTTGAGCTGTCGGGAGGGGGTGGTTGGTG	0.329	
36	ACTGTGGGCGGGGAATAGGTGTGTCGGGGTAGGGGACGCT	0.327	
37	TGTGGGCGGGGGTTAACGTCTCCTGTCGGGTGGGGGGCTG	0.301	
38	AGTGGGTGGGGGTGATCAATTGTCCTGTCGGGCGGGGGTG	0.295	
39	CGTGGGTGGGGGCCGTTTACTTTTGTCGGGGAAGGGGGTG	0.292	
40	ATGTGGGGGAACTGTTGGGGGTTATGGCTGTCGGGCGGGG	0.282	
41	TGTGGGCGGGGTGTAAGTCTGTCGGGGCCGGGGACGTCGA	0.274	
42	ATCGGGTTTGGCATTGGGCCTGGCAGGATGGGCCTGTGAG	0.259	
43	ATTCGGTGGGTGGGGTCGGCGCTGTTTGTCGGGGTCCGGG	0.257	
44	TACATTGGTGGGTGGGGTATTTTGTGCTGTCGGGGTCGGG	0.244	
45	TGTGGGCGGGGGGAATAATTGCAGCTGTCGGGCGGGGGTG	0.242	
46	TCGTGGGCGGGGGTCGGCGGTTTCCTTCTGTCGGGCGGGG	0.217	
47	TGTGTGGGGGTCTCCGGGGGTTAGTTTCTGTCGGGCGGGG	0.211	
48	TGTGGGTGGGGGTTTCTCTGTCGGGGGTGCCGGGGTCGTG	0.209	
49	GCGGGTGGGGGAACATCACGCACTGTCGGGGCCGGGGGTG	0.208	
50	GCGGGTGGGGGTTGGAGGTCTTTTCTCTGTCGGGAGGGGG	0.196	10.2 ± 2.6
51	CGCGGGTGGGGGGTGTATGTATTCTTGTCGGGCGGGGGTG	0.193	
52	GTAGGGTTTGGCTCCGGGCCTGGCGTCGGTCGTCTCTCGC	0.192	
53	TGTGGGCGGGGGATCAGTCAGAAGTTGTCGGGCGGGGGTG	0.179	
54	ATTCTCGTGTGTGGGTGGGGTAATATCTGTCGGGGTTGGG	0.172	
55	CTCATGTGGGCGGGGGATGTATTATTTTGTCGGGGTTGGG	0.171	
56	CGGGTTGCGACGTGGGATGCTGTTTGGTTTGGTCTGGTTG	0.170	
57	TGCGGGTGGGGGTCGGTCGGCATCTGTCTGTCGGGTGGGG	0.168	
58	CGCGGATTGTGCGTTTACTGGGTTTGGCTCCGGGCCTGGC	0.163	
59	TGTATTGTGGGTGGGGATTTCGATCTGTCGGGGTATGGG	0.156	
60	ACTGGGTTTGGCTCCGGGCCTGGCGTGGGATGGTGTCCGT	0.155	
61	TGGTGGGCGGGGTTGTACGTTTGTGCTTTTGTCGGGTGGG	0.150	
62	GCACAGTACCTACTGGTGCGATGTACTCCGCCTAACGTGG	0.144	
63	CTCGTGGGCGGGGGCTAGATAGGTTTGTCGGGCGGGGGTG	0.140	
64	TCGTGGGCGGGGGTTTGTATTGGCGTGTCGGGAGGGGGTG	0.138	
65	TAGTGGGCGGGGGCTATTTCTTGTCGGGGTTTGGGAGGTG	0.131	
66	TACTGTGGGCGGGGTATCATGGTCTGTCGGGGCTCGGG	0.126	

67	TGGGTTTGGCTGCGGGCCTGGCGGAGGTCGATGAGTAGCG	0.123	
68	TGTCGGGGATGGGGCTTTATATTGCAATGTGGGGCCGGGG	0.121	
69	CCTAGGTTGTCGGGTGGTTGTCATTGTGGGTGGGAAAGCC	0.117	
70	CCACCGCATATTAAGTGTGCAATAACTCCACCTGGTGTTG	0.117	
71	CTGGTGGGTCGGGGGCTGAAGACTGTCGGGTGGGGGTTG	0.114	
72	TGTGGGTGGGGGTTATATCTGTCGGGGGTTACGGGGGCCT	0.113	
73	ATCGGTGGGTGGGGGGTAAATGCTGTCGTGGTGGGGGCTG	0.112	
74	TGTGGGGTTGGGGTTGTCTATTTGTCACTGTCGGGTGGGG	0.111	
75	TGTGGGTGGGGTTGGTGGTTGGTATTCTTTGTCGGGCGGG	0.110	
76	AGCAGTGGGTGGGGGTTAAAACTTTTCTGTCGGGGCCGGG	0.107	
77	TAGGGATTGGCATTGGGCCTGGCATGGGGACGCGAGTGGTC	0.104	
78	TGTGGGCGTGGGGCGTTTTGATATGTCGGGGTCGGGGGTG	0.100	
79	TCGGTGGGGATCGGGGGCCATCTTTTCTGTCGGGCGGGGT	0.099	
80	ATGTGGGGTCGGGGTCTGGTCTACACTGTCGGGGTCCGGG	0.097	
81	TGTGGGTGGGGTTAGTTCTCATGTTTCGTTGTCGGGCGGG	0.096	
82	AGTGGGCGGGGGCTCTATAGTGATTGTCGGGTGGGGGTTG	0.093	
83	CCGTGGGTGGGTGCTGTTTTGTCGGGCGGGGGCGGAGGTG	0.093	
84	CGTGGGCGGGGGCTGGTAAGTAGATTTCTGTCGGGTGGGG	0.090	
85	TAGGGTTTGGCATTGGGTCTGGCATAGAGCAAGTCGCTGT	0.090	
86	TGCAAGTGGGGTTTTCGGGGAGGTTATGTCGGGTGGGGTG	0.089	
87	TGCGGGGGATTGTTGGGGGTCAAGCTGTCGGGTGGGGGTT	0.089	
88	AGTGTGGTGGGGTAATAACTGTCGGGGTTGGGGTGGTGCC	0.086	
89	GGCCTATTGTGGGTGGGGAAAAGGTTTGTCGGGGTCGGGG	0.083	
90	GGGGATTGGCTTTGGGCCTGGCAAGGGATTTTACATGCGT	0.081	
91	ATGCGGGTGGGGGCCATAGTTCTCGTGTCGGGCGGGGGTG	0.080	
92	GCTGGTGGGTTGGGGGCATTTATCTGTCGGGCGGGGGTTG	0.080	
93	CATGTGGGCGGGGGCTTGTACTGGTTGTCGGGTGGGGGCG	0.078	
94	TAGCTGTGGGGCATGGGGAGAAAATGTTGTCGGGTGGGGT	0.076	
95	CGGTGGGATGGGGGCTCCAGATATGTCGGGGTTGGGGGTG	0.074	
96	ACGGTGGGAGGGGGGTATTGCAGTTGTCGGGTGGGGGCCG	0.072	
97	GTCGCGGGTTTGGCATCGGGTCTGGCGGCGCGGGTGCCTC	0.071	
98	ACTGCGGGAGGGGGTGCGAACTTTGTCGGGGCTGGGGGTG	0.071	
99	TGGGATTGGCTCCGGGCCTGGCGTGGTCGGACTTTTTGGC	0.070	
100	TAGTGGGCGGGGGCGATTACCTATTTGTCGGGCGGGGGTG	0.068	
439	GCGAACGGATTGGTTGGACTGGTTGGGTGAGTCGGTAGGC	0.007	36.9 ± 5.5

[a]: Each sequence contains primer regions of TTACGTCAAGGTGTCACTCC and GAAGCATCTCTTTGGCGTG at the 5' end and 3' end, respectively.

Table S4. Sequence and K_d values of published DNA aptamers.

Name	Sequences (5'→3')	K_d (nM)[a]	Size (nt)	Target	Ref.
Aptamer-1	ATCCAGAGTG ACGCAGCATC GAGTGGCTTG TTTGTAATGT AGGGTTCCGG TCGTGGGTTG GACACGGTGG CTTAGT	S: 28; Virus: 6.05 ± 2.06	76	RBD	Xiaohui Liu *et al*[1]
Aptamer-2	ATCCAGAGTG ACGCAGCAAT TACCGATGGC TTGTTTGTAA TGTAGGGTTC CGTCGGATTG GACACGGTGG CTTAGT	S: 27; Virus: 6.95 ± 1.10	76		
Aptamer-6	ATCCAGAGTG ACGCAGCAGG GCTTGGGTTG GGAATAAGGA TGTGGGAGGC GGCGAACATG GACACGGTGG CTTAGT	S: 24; Virus: 7.52 ± 3.20	76		
SP5	GGGAGAGGAG GGAGATAGAT ATCAACCATG GTAGGTATTG CTTGGTAGGG ATAGTGGGCT TGATGTTTCG TGGATGCCAC AGGAC	S: 14.7 ± 0.8	85	Trimerized Spike protein of SARS-CoV-2	Anton Schmitz *et al*[2]
SP6	GGGAGAGGAG GGAGATAGAT ATCAACCCAT GGTAGGTATT GCTTGGTAGG GATAGTGGGC TTGATGTTTC GTGGATGCCA CAGGAC	S: 13.9 ± 0.6	85		
SP6.34	CCCATGGTAG GTATTGCTTG GTAGGGATAG TGGG	NA	34		
SP7	GGGAGAGGAG GGAGATAGAT ATCAAAGGAG GGTAGGTAGT GCTTGGTAGG GAAACTCCGC CGATTTTTCG TGGATGCCAC AGGAC	S: 13.1 ± 3.8	85		
CoV2-2	ATCCAGAGTG ACGCAGCAGG GATGGGCTCC GGGCTACTGG CGAGGCTTCG GAACAACCGG ACACGGTGGC TTAGTA	NA	76	RBD	Miao Sun *et al*[3]
CoV2-6	ATCCAGAGTG ACGCAGCACC CAAGAACAAG GACTGCTTAG GATTGCGATA GGTTCGGGGG ACACGGTGGC TTAGTA	RBD: 84.64 ± 10.64	76		
CoV2-6C3	CGCAGCACCC AAGAACAAGG ACTGCTTAGG ATTGCGATAG GTTCGG	RBD: 44.78 ± 9.97	46		
cb-CoV2-6C3	CGTAAATCAG TCACGCAGCA CCCAAGAACA AGGACTGCTT AGGATTGCGA TAGGTTCGGT GACTGATTTA CGCGCAGCAC CCAAGAACAA GGACTGCTTA GGATTGCGAT AGGTTCGG	RBD: 0.13 ± 0.04	118		
CoV2-RBD-1	ATCCAGAGTG ACGCAGCACC GACCTTGTGC TTTGGGAGTG CTGGTCCAAG GGCGTTAATG GACACGGTGG CTTAGT	RBD: 3.1 ± 0.6	76	RBD	Yanling Song *et al*[4]
CoV2-RBD-4	ATCCAGAGTG ACGCAGCATT TCATCGGGTC CAAAAGGGGC TGCTCGGGAT TGCGGATATG GACACGGTGG CTTAGT	RBD: 13.6 ± 3.4	76		
CoV2-RBD-1C	CAGCACCGAC CTTGTGCTTT GGGAGTGCTG GTCCAAGGGC GTTAATGGAC A	RBD: 5.8 ± 0.8	51		
CoV2-RBD-4C	ATCCAGAGTG ACGCAGCATT TCATCGGGTC CAAAAGGGGC TGCTCGGGAT TGCGGATATG GACACGT	RBD: 19.9 ± 2.6	67		
Apt-S-79s	GTCTTGCGGG GCGGCGGGTT GAGAGGA	NA	27	S1	Ran Liu *et al*[5]
Apt-S-268s	GGGGTGGGGT AGTGGTATGG AGCG	NA	24		

[a] S: Spike protein; Virus: S/RBD-virus mimics; RBD: the receptor-binding domain (RBD) of the SARS-CoV-2 spike protein; NA: no K_d value reported.

[1] X. Liu, Y.-l. Wang, J. Wu, J. Qi, Z. Zeng, Q. Wan, Z. Chen, P. Manandhar, X. Fu, X. Zhang, *Angew. Chem. Int. Ed.* **2021**, *doi.org/10.1002/anie.202100345*.

[2] G. Mayer, A. Schmitz, A. Weber, M. Bayin, S. Breuers, V. Fieberg, M. Famulok, *Angew. Chem. Int. Ed.* **2021**, *doi.org/10.1002/anie.202100316*.

[3] M. Sun, S. Liu, X. Wei, S. Wan, M. Huang, T. Song, Y. Lu, X. Weng, Z. Lin, H. Chen, *Angew. Chem. Int. Ed.* **2021**, *doi.org/10.1002/anie.202100225*.

[4] Y. Song, J. Song, X. Wei, M. Huang, M. Sun, L. Zhu, B. Lin, H. Shen, Z. Zhu, C. Yang, *Anal. Chem.* **2020**, *92*, 9895-9900.

[5] R. Liu, L. He, Y. Hu, Z. Luo, J. Zhang, *Chemical Science* **2020**, *11*, 12157-12164.

A

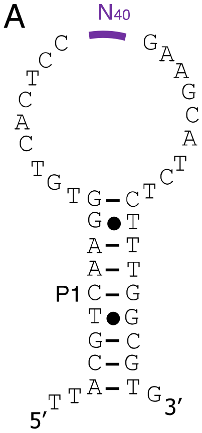

B

FP1 (Forward primer 1)
TTACGTCAAG GTGTCACTCC

RP1 (Reverse primer 1)
CACGCCAAAG AGATGCTTC

RP2 (Reverse primer 2)
TTTTTTTTTT TTTTTTTTTT-L-

CACGCCAAAG AGATGCTTC

Figure S1. The design of the DNA library. (A) The pre-engineered secondary structure of the DNA library. (B) The sequences of the forward and reverse primers for the amplification of the DNA library by PCR.

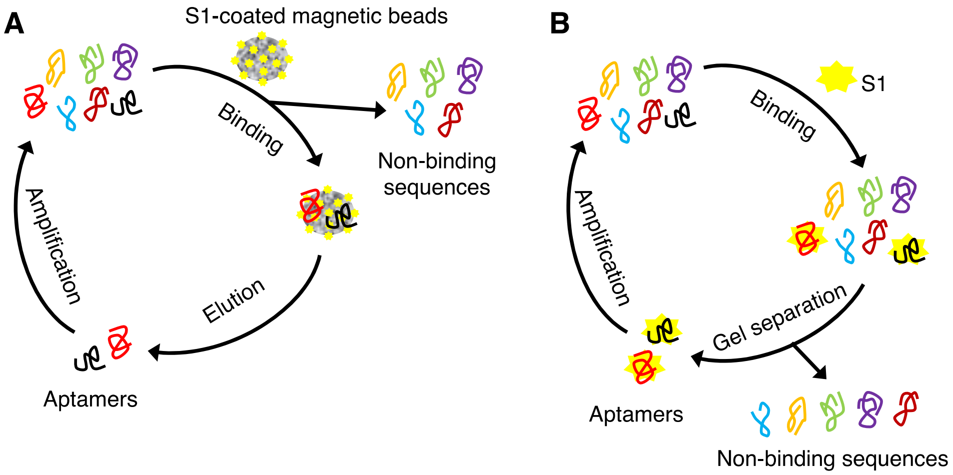

Figure S2. *In vitro* selection schematic used for the isolation of DNA aptamers for the S1 protein (S1) of SARS-CoV-2. (A) Bead-based selection. The selection began with a library containing ~6×10^{14} unique sequences. The library was incubated with S1-coated magnetic beads to retain S1 binding sequences, which were eluted and amplified by PCR to produce an enriched pool for the next round of selection. The first three cycles were performed with this method. (B) Gel based selection. For selection cycles 4-13, the DNA pool was incubated with free S1, followed by native polyacrylamide gel electrophoresis to isolate the DNA/S1 complexes which have retarded gel mobility. The complex band was cut out from the gel, eluted and amplified by PCR to produce a new pool for the next round of selection.

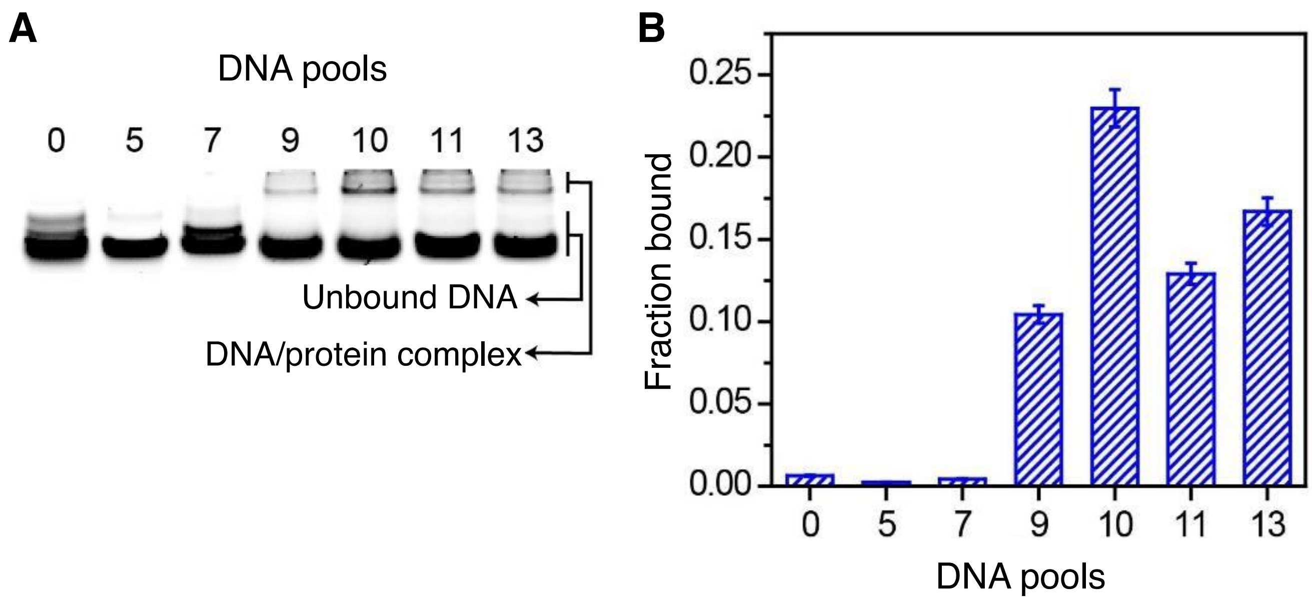

Figure S3. Assessment of binding of selected enriched DNA pools. (A) EMSA and (B) Fraction bound of pools 0, 5, 7, 9, 10, 11 and 13. FAM-labelled DNA (25 nM) and S1 protein (250 nM) were used for the binding analysis.

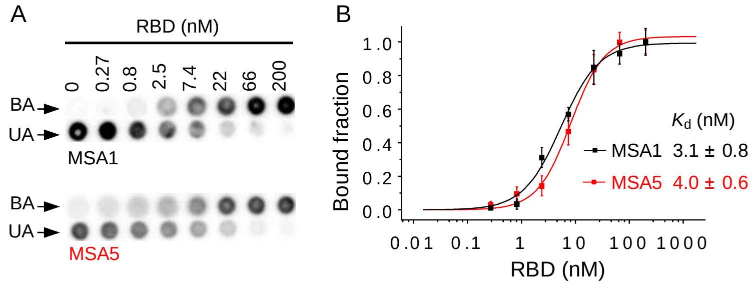

Figure S4. Assessment of MSA1 and MSA5 for RBD binding. (A) Dot blot results of MSA1 and MSA5 binding to the RBD of the spike protein of SARS-CoV-2. BA: bound aptamer; UA: unbound aptamer. (B) Binding curves used to derive the K_d values.

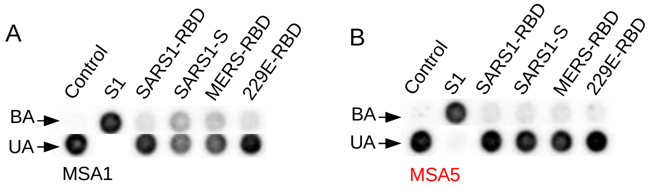

Figure S5. Dot blot results of (A) MSA1 and (B) MSA5 for binding to the S1 protein of SARS-CoV-2 and control proteins that include the RBD and the spike (S) protein of SARS-CoV1, the RBD of MERS, and the RBD of HCoV-229E. 50 nM proteins were used for the tests.

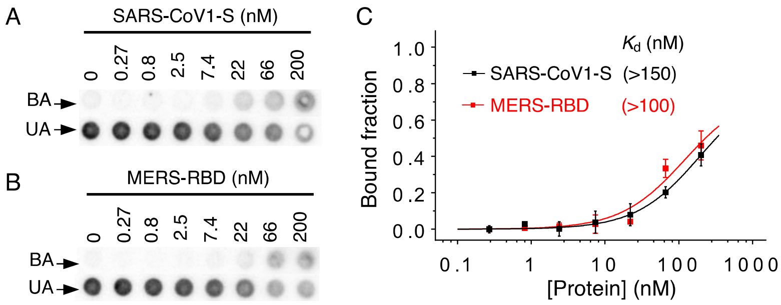

Figure S6. Dot blot results of MSA1 for binding to (A) the spike (S) protein of SARS-CoV-1 and (B) the RBD of MERS. BA: bound aptamer; UA: unbound aptamer. (C) Binding curves used to evaluate the K_d values.

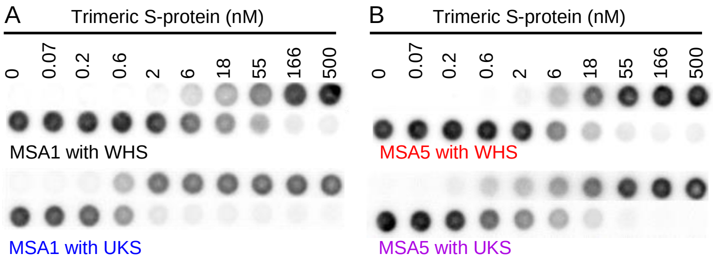

Figure S7. Representative dot blot results showing binding of (A) MSA1 and (B) MSA5 to the trimeric S protein of the wild-type Wuhan variant (WHS) and the UK B.1.1.7 variant (UKS) of SARS-CoV-2.

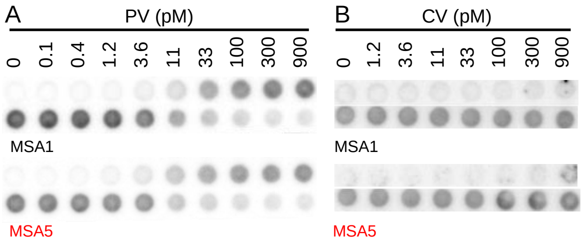

Figure S8. Dot blot results of MSA1 and MSA5 for binding to (A) a pseudotyped lentivirus (PV) that was engineered to display the wild-type S-protein of SARS-CoV-2 and (B) the same lentivirus that lacks the S-protein (CV).

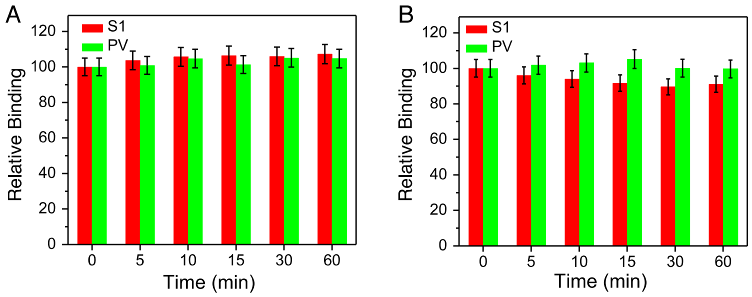

Figure S9. Relative binding activity of (A) MSA1 and (B) MSA5 to S1 protein and pseudotyped virus of SARS-CoV-2.

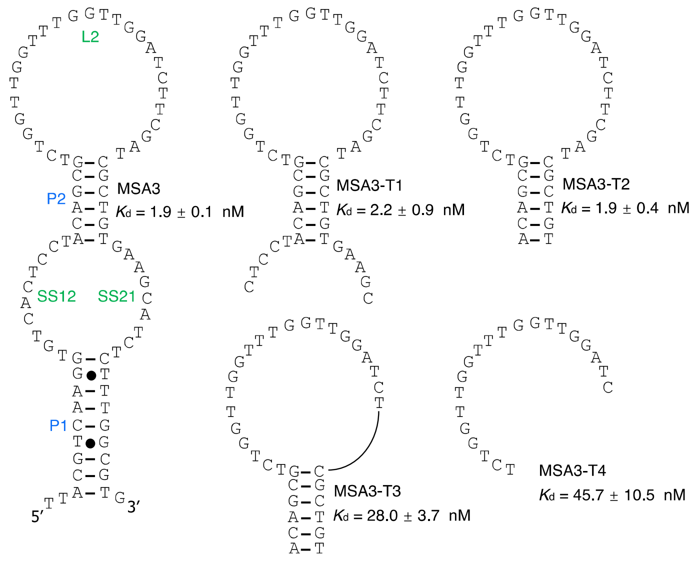

Figure S10. The predicted secondary structure of MSA3 and the binding activity of its truncation mutants.

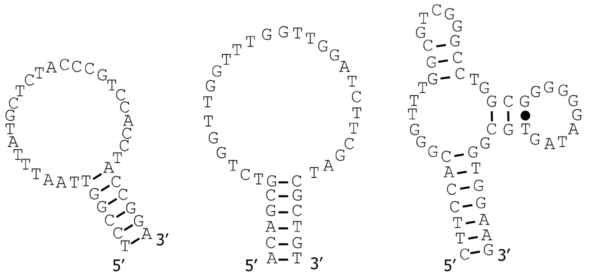

MSA1-T3 (37 nt)
K_d = 3.1 ± 0.4 nM

MSA3-T2 (39 nt)
K_d = 1.9 ± 0.4 nM

MSA5-T4 (49 nt)
K_d = 6.3 ± 0.8 nM

Figure S11. Comparison of the hairpin structures of the minimized mutants of MSA1, MSA3 and MSA5.

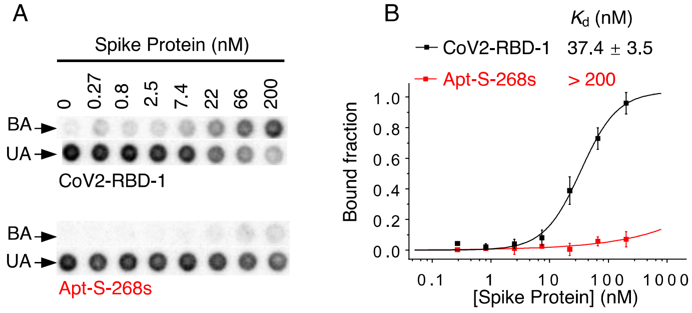

Figure S12. (A) Dot blot results of two reported aptamers, CoV2-RBD-1 and Apt-S-268s, for binding to the spike protein of SARS-CoV-2. BA: bound aptamer; UA: unbound aptamer. (B) Binding curves used to derive the K_d values.

A

	Cross Comparison BLAST Score Heat Map	MSA1	MSA2	MSA3	MSA4	MSA5	MSA6	MSA7	MSA8	MSA9	MSA10	Zhu1	Zhu3	Mayer_SP5	CoV2-2	CoV2-6	CoV2-RBD-1C	CoV2-RBD-4C	Apt-S-79s	Apt-S-268s
		Top 10 Aptamers in MSA Selection										Published Spike Aptamers								
Top 10 Aptamers in MSA Selection	MSA1	75				12.2						14								
	MSA2		75	12.2	12.2	15.9		15.9		21.4		14	15.9	12.2			12.2	14	19.6	12.2
	MSA3		14	75	23.3	12.2	14	12.2	14	12.2	28.8							12.2		10.4
	MSA4		12.2	23.3	75	15.9	14	12.2	14	12.2	32.5			17.7	12.2				12.2	12.2
	MSA5	12.2	15.9		15.9	75		23.3		23.3		12.2	15.9	12.2				12.2	14	10.4
	MSA6			14	14		75		73.1		14		12.2							
	MSA7		15.9		12.2	23.3		75		23.3		14	12.2			15.9		12.2		14
	MSA8			14	14		73.1		75		14		12.2							
	MSA9		21.4		12.2	23.3		23.3		73.1		12.2	14					12.2		15.9
	MSA10			28.8	32.5		14		14		75	12.2			21.4		12.2	12.2	14	
Published Spike Aptamers	Zhu1	14	14			12.2		14		12.2	12.2	75	12.2	12.2	12.2					10.4
	Zhu2		15.9			15.9	12.2	12.2	12.2	14		12.2	75	14		12.2	12.2		14	10.4
	Mayer_SP5		12.2		17.7	12.2						12.2	14	75	12.2					14
	CoV2-2				12.2						21.4	12.2		12.2	76.8	12.2				
	CoV2-6							15.9					12.2		12.2	75		17.7		
	CoV2-RBD-1C		12.2								12.2		12.2				75	14		
	CoV2-RBD-4C		14	12.2		12.2		12.2		12.2	12.2					17.7	14	75	12.2	
	Apt-S-79s		19.6		12.2	14					14		14					12.2	51	
	Apt-S-268s		12.2		12.2			14		15.9				14						45.4

>=50	40	30	20	10	No Hit	BLAST Score (bits)

B

```
Apt-S-79s vs. MSA2 - Score = 19.6 bits, Expect = 0.042
MSA2         GTCTTGTCGGGGTCGG-GGGT
             |||||| ||||| ||| ||||
Apt-S-79s    GTCTTG-CGGGG-CGGCGGGT
```

CoV2-2 vs. MSA10 - Score = 21.4 bits (11), Expect = 0.009

```
MSA10    GGGTTTGGCTCCGGGC--CTGGCG
         |||  | ||||||||||  ||||||
CoV2-2   GGGATGGGCTCCGGGCTACTGGCG
```

Figure S13. Nucleotide BLAST comparison of MSA1-10 aptamers identified in this study and a selection of published spike aptamer sequences. (A) Nucleotide BLAST scores for all sequence pairs where a hit was reported. Sequences of high similarity generate high scores indicated in dark red shading. Numerical scores of sequence pairs where alignments were found are reported, empty squares indicate no Nucleotide BLAST alignment was found. High alignment scores along the diagonal represent self-alignment. High scores along a column or

row indicate similarity of the sequence to other sequences in the dataset. BLASTn algorithm was run with parameters word size = 5 and strand = plus/plus. Heatmap is coloured based on bit-score intervals. (B) Local alignment of the two most significant hits observed between MSA1-10 aptamers and published aptamers (Apt-S-79s vs. MSA2 and CoV2-2 vs. MSA10). Alignment is observed between G-rich regions of both aptamer sequences. Due to the high frequency of G-rich sequences often observed in in vitro selection, the significance of an alignment in these regions may be over-estimated.
